# VP2-targeted sandwich ELISA (sELISA) enables direct detection of Senecavirus A (SVA)

**DOI:** 10.1128/jvi.00571-26

**Published:** 2026-05-12

**Authors:** Robert M. Hnasko, Alice V. Lin, Jeffery A. McGarvey

**Affiliations:** 1 Western Regional Research Center (WRRC) Produce Safety and Microbiology Research Unit (PSM), United States Department of Agriculture (USDA), Agricultural Research Service (ARS), Pacific West Area (PWA) 551257 , Albany, California, USA; 2 USDA, ARS, PWA, WRRC, FTDP 1096 , Albany, California, USA; The University of Texas Southwestern Medical Center, Dallas, Texas, USA

**Keywords:** Senecavirus A, VP2, diagnostics, monoclonal antibodies, immunoassay

## Abstract

Senecavirus A (SVA) is an emerging swine virus that causes vesicular lesions clinically indistinguishable from other high-consequence transboundary viral vesicular diseases, creating significant diagnostic and economic challenges. Rapid and specific detection of SVA is, therefore, critical for supporting foreign animal disease (FAD) investigations. In this study, we generated and characterized monoclonal antibodies (MAbs) targeting the SVA VP2 capsid protein, and assessed their performance in multiple immunoassay formats. Two MAbs, 2D1 and 7B3, demonstrated strong and specific binding to both recombinant VP2 and native SVA. Using these antibodies, we developed a sandwich ELISA (sELISA) capable of direct viral detection with a sensitivity of 60 pg/mL. This assay detects contemporary SVA strains and provides a rapid, high-sensitivity platform suitable for differential disease diagnosis of idiopathic vesicular disease. These VP2-directed immunoassays offer valuable tools to enhance SVA surveillance and streamline FAD investigative workflows.

**IMPORTANCE:**

Senecavirus A (SVA) produces vesicular lesions that are clinically indistinguishable from foot-and-mouth disease and other high-consequence transboundary viruses, making rapid and accurate differentiation essential during foreign animal disease (FAD) investigations. These investigations impose substantial operational and economic burdens, including movement restrictions and diagnostic delays caused by the lack of rapid, field-deployable testing tools. Current surveillance relies heavily on laboratory-based molecular assays, underscoring the acute need for a rapid, pen-side method capable of direct SVA detection. The monoclonal antibodies developed in this study target conserved, surface-exposed VP2 on the SVA capsid, enabling the first sandwich ELISA capable of detecting native virus with high sensitivity across circulating strains. This diagnostic platform provides a fast, inexpensive tool with the potential to improve differential diagnosis, accelerate FAD investigations, and reduce the operational disruptions associated with SVA-related outbreaks.

## INTRODUCTION

Senecavirus A (SVA) is a non-enveloped positive-sense single-stranded RNA virus with swine as its natural host. SVA is the only member of the genus *Senecavirus* in the family Picornaviridae and its genome contains a single open reading frame (ORF) encoding four structural proteins (VP1-4) and eight non-structural proteins (L, 2A-C, and 3A-D) typical of this viral family (1, 2). The four structural proteins facilitate viral assembly and the formation of an icosahedral capsid of ~30 nm in diameter that interacts with host cells (3).

SVA is endemic in the Americas and afflicts pigs with vesicular lesions of the snout and coronary bands (4). Infection in adult pigs has a low rate of mortality, but a high morbidity, with clinical disease resolving by 14 days post-infection in most animals (5). There is no vaccine, but the disease is self-limiting, and palliative care of afflicted animals is the only treatment (6). SVA has a significant economic and logistical impact on the swine industry given its identical clinical presentation to other serious high-consequence transboundary viral vesicular diseases such as foot-and-mouth disease (FMD), swine vesicular disease (SVD), vesicular stomatitis (VS), and vesicular exanthema of swine (VES) (7). There is a high prevalence of SVA in tested pig populations in North America (8, 9), and any livestock with vesicular disease symptoms requires investigation to rule out FMD viral infection, as recommended by the World Organization for Animal Health (WOAH) (10, 11). The initiation of foreign animal disease (FAD) investigations of animals presenting clinical vesicular disease frequently involves market-weight swine, resulting in temporary restrictions on animal movement and disruption of market supply chains. There remains an acute need for improved cost-efficient tests to facilitate detection of SVA to aid in differential disease diagnosis and expedite FAD investigations.

The highest concentration of SVA nucleic acid is localized to vesicular lesions (12–14), and detection methods include RT-PCR, serology, and viral propagation in susceptible cell lines (15). Although monoclonal antibodies have been generated against SVA capsid VP proteins, their utility in immunoassay applications has been limited to indirect detection methods (16–19). The VP2 protein is exposed on the outer surface of the viral capsid and is highly conserved among different SVA strains (20, 21). Although the VP proteins share structural homologies with other picornaviruses, the primary structure of SVA VP2 shares minimal sequence identities to homologous counterparts (22). In this manuscript, we report the generation of monoclonal antibodies (MAbs) directed against recombinant VP2 protein of SVA, characterize SVA binding of two anti-VP2 MAbs, and describe their development into the first sandwich ELISA (sELISA) for the direct detection of SVA. These SVA immunoassays will enhance SVA detection in animals exhibiting symptoms of idiopathic vesicular disease and facilitate rapid investigative reporting.

## MATERIALS AND METHODS

### Recombinant Senecavirus A (SVA) VP2 protein

A synthetic nucleotide construct of 918 base pairs was inserted into the bacterial plasmid pET30a using enzyme cleavage sites 5′-NdeI (CATATG) and 3′-HindIII (AAGCTT), encoding a 306 amino acid VP2 protein of the SVA virus SD15-26 (NCBI: APY18927; amino acid residues 151–434 of the 2181 amino acid polypeptide), which includes an N-terminal methionine and a modified C-terminus (polyanionic tag GDDD triplet, three lysine, and six histidine residues). *Escherichia coli* BL21-DE3 competent cells were transformed with the VP2-pET30a plasmid, and a clonal colony inoculated into LB medium containing kanamycin and then cultured at 37°C at 200 RPM to a cell density of 0.7 OD at 600 nm. IPTG (0.5 mM) was used for plasmid induction for 16 h at 15°C, and bacterial pellets were extracted in PBS with imidazole, purified by nickel-affinity chromatography (HisPur Ni-NTA resin; Thermo Fisher), and fractions dialyzed into PBS (20K MWCO). Recombinant VP2 protein expression was evaluated by SDS-PAGE and Western blot using an anti-HIS antibody (GenScript A00186), reporting a ~36 kDa protein.

### Immunization and hybridoma fusions

Female Balb/cByJ mice, 6 weeks of age (The Jackson Laboratory, ME), were inoculated by intraperitoneal injection with 5 µg of purified recombinant SVA VP2 protein in a 100 µL emulsion with TiterMax Gold adjuvant (T2684; Sigma, MO) and 2× PBS at equal volume. Mice were inoculated 4× every 10 days, with the final boost containing no adjuvant 3 days prior to cell fusion. As previously described (23, 24), mouse splenocytes were harvested and chemically fused to a subclone of the MPC11–45.6TG1.7 myeloma cell line (2:1 ratio) using stepwise dilution of poly(ethylene glycol) (PEG 3–3.7K, Sigma) in serum-free medium. Fused cells were then diluted into tissue culture flasks containing Iscove’s medium with 10% heat-inactivated fetal bovine serum (FBS) and HAT (hypoxanthine-aminopterin-thymidine) for 3 days at 37°C in 5% CO_2_. After 3 days, cells were centrifuged over Histopaque-1077 (Sigma), and viable cells were collected, counted, and plated at limiting dilution in tissue culture (TC) microplates. Cell colony formation occurred between 10 and 15 days, and cell-conditioned media (CM) from each microwell were evaluated by indirect enzyme-linked immunosorbent assay (iELISA) using recombinant VP2 protein immobilized on high-binding (HB) black microplates (Greiner Bio-One, NC), with VP2 antibody detection in CM by luminometry (Victor X3; Perkin-Elmer, MA) using a goat anti-mouse IgG gamma-chain horseradish peroxidase (HRP) conjugate (1:10K, AP503P; Sigma) and a chemiluminescent substrate (Ultra ECL; Neogen, MI).

### Hybridoma cell lines and anti-VP2 monoclonal antibodies (MAbs)

Microplate wells containing hybridoma cells with CM positive for VP2 detection by iELISA were expanded into 24-well TC microplates for 2 days, and CM was evaluated by capture ELISA (cELISA). Briefly, HB microplates were coated with 2 µg/mL donkey anti-mouse-Fc IgG (H + L) (AffiniPure 715-005-150; Jackson ImmunoResearch, PA), washed in TBST, and then incubated with hybridoma CM. Microplate wells were washed, incubated with a biotinylated recombinant VP2 protein (Lightning-Link Fast, Type A; Abcam, MA), and antibody-captured VP2 protein was detected by luminometry (Victor X3) using an avidin-HRP (1:30K, A7419; Sigma), followed by a chemiluminescent substrate (Ultra ECL). Hybridoma cells with CM positive for binding in both iELISA and cELISA were subjected to cell cloning by limiting dilution and repeated evaluation by ELISA, resulting in a cohort of clonal hybridoma cell lines producing anti-VP2 MAbs. During limiting dilution, hybridoma cells were grown in Iscove’s medium containing 10% FBS and HT, with the final clonal cell expanded in Iscove’s with only 10% FBS as a cell line. Mouse hybridoma cell lines producing anti-VP2 MAbs were preserved by freezing in 10% DMSO (Hybri-Max D2650; Sigma) in FBS and stored in liquid nitrogen for recall. A cohort of 14 hybridoma cell lines was established, and their MAbs were shown to bind recombinant VP2 (Table S2). Only the 2D1 and 7B3 MAb pair were determined to effectively bind the SVA SD15-26 virus. These anti-VP2 MAbs are both IgG isotypes (subclass 1) with kappa light chains.

### Live cell imaging

A Leica inverted phase-contrast DMI4000B microscope with an attached MC170 HD digital camera was used to collect phase-contrast photomicrographs of live cells. Human lung NCI-H1299 epithelial-like cells (CRL-5803; ATCC, VA) were grown in clear 6-well tissue culture plates to 70% confluence in Iscove’s medium containing 10% FBS and either mock- or SVA SD15-26-infected conditions for 48 h at 37°C and 5% CO_2_ prior to imaging.

### MTT assay

Water-soluble thiazolyl blue tetrazolium bromide (MTT) was prepared as a 5 mg/mL stock in serum-free Iscove’s medium and stored at 4°C in the dark. NCI-H1299 cells were seeded at a uniform density of 20K cells per well in flat-bottom clear TC 96-well microplates and allowed to attach overnight in Iscove’s medium with 10% FBS at 37°C in 5% CO_2_. The following day, culture medium was aspirated and replaced with 100 µL of culture medium consisting of a logarithmic dilution of SVA SD15-26-infected cell-conditioned media (CM) collected 24 h and 48 h post-infection and filtered (0.22 µm PES). After 48 h of incubation, 50 µL of MTT stock medium was added directly to each microplate well and incubated at 37°C at 5% CO_2_ for 3 h. Purple formazan crystals are visible under a microscope in metabolically active cells. The MTT medium was aspirated, and 150 µL of acidic alcohol (4 mM HCl in isopropanol with 0.1% NP40) was added to each well. The microplate was shaken for 15 min at room temperature to dissolve the formazan crystals, along with manual pipetting. The MTT reaction product was analyzed by absorbance spectroscopy at 560 nm (Victor X3) and was expressed as mean ± SEM (*N* = 3).

### Western blot

Recombinant SVA VP2 protein was diluted in LDS sample buffer (lithium dodecyl sulfate, Coomassie G250, phenol red, and glycerol at pH 8.4), treated with the reducing agent TCEP (tris(2-carboxyethyl)phosphine hydrochloride) to 10 mM (+), and heated to 70°C for 10 min. VP2 protein was subject to electrophoresis through 4–12% Bis-Tris polyacrylamide gels (NuPAGE; Thermo Fisher) using MOPS SDS running buffer (pH 7.7). A color protein marker (Precision Plus Protein Dual Color Standard; Bio-Rad, CA) was used to estimate protein molecular weight (MW) in kilodaltons (kDa). Gels were either stained with colloidal Coomassie (ReadyBlue; Sigma) or proteins transferred to nitrocellulose membranes and subjected to Western blotting using anti-VP2 MAbs 2D1- and 7B3-HRP conjugates (Lightning-Link; Abcam, MA) at 1 µg/mL with a chemiluminescent substrate (PicoECL; Thermo Fisher). Images were acquired using an Alpha-Innotech FluorChem HD2 (ProteinSimple, CA) and shown inverted, with proteins resolved as dark bands. The VP2 protein migrates at ~36 kDa.

### Direct ELISA (dELISA)

Purified recombinant SVA VP2 protein was subjected to logarithmic dilutions in 0.2 M carbonate-bicarbonate buffer (pH 9.4) and immobilized on black HB microplates for 1 h at room temperature (RT). Microplates were washed in TBST (pH 7.2), blocked in 2% IgG-free BSA (Jackson ImmunoResearch, PA), and incubated for 1 h at RT with HRP-conjugated (Lightning-Link) primary MAb (2D1 or 7B3; 1 µg/mL). VP2 detection was determined by luminometry (Victor X3) using a chemiluminescent substrate (Ultra ECL). Data are reported in counts per second (CPS), and a no-antigen negative control was used for statistical comparison (*N* = 4).

### Sandwich ELISA (sELISA)

The 7B3 or 2D1 MAb was immobilized at 2 µg/mL in 0.2 M carbonate-bicarbonate buffer (pH 9.4) on black HB 96-well microplates for 1 h, washed in TBST, blocked for 1 h in 2% IgG-free BSA TBST, then incubated with dilutions of VP2 or SVA-infected NCI-H1299 cell CM overnight at RT. After TBST washing, either the 7B3- or 2D1-HRP MAb conjugate (1 µg/mL in TBST with 0.2% BSA) was added for 1 h, washed again in TBST before adding a chemiluminescent substrate (Ultra ECL) with detection by luminometry (Victor X3) reported in CPS after 3 min, or a blue colorimetric 3,3′,5,5′-tetramethylbenzidine (TMB) substrate (K-blue Advanced; Neogen), reporting spectroscopic absorbance (Victor X3) at 560 nm after 10 min.

### Immunofluorescent microscopy

NCI-H1299 cells grown in 6-well TC plates were infected with SVA SD15-26 for either 24 h or 48 h. A cytospin instrument (Shandon, MA) was used to concentrate 0.5 mL of suspended cells in culture medium by centrifugation at 900 × *g* for 3 min onto coated cytoslides (Epredia, MI). Slides were washed in PBS (pH 7.2), fixed for 3 min in 10% neutral buffered formalin, washed in water, dried on a hot plate for 1 h at 40°C, and stored at 4°C until use. The 7B3 and 2D1 anti-VP2 MAbs were conjugated to fluorescein isothiocyanate (Lightning-Link FITC, Abcam) and diluted to 150 µg/mL in PBS with 0.1% Triton X-100 (PBX) and 1% FBS. Slides were washed in PBX, blocked in 2% IgG-free BSA for >1 h, then incubated with MAb conjugates for 3 h at RT, washed in PBX, incubated with DAPI (4′,6-diamidino-2-phenylindole) nuclear stain (1:20K in PBX), washed in water, and coverslips were applied with Histomount (Thermo Fisher). Micrographs were taken using a Leica SP5 confocal microscope with excitation by an argon 488 nm (FITC) and 405 nm (DAPI) laser, using 20× and 40× objectives. Mock-infected cells (no virus) served as a negative control.

### Antibody epitope mapping

VP2 amino acid residues 151–434 from the Senecavirus A (SVA) protein sequence (ALF40134.1) was used to generate a series of 28 sequential peptides of 15 amino acids containing an N-terminal Ahx-biotin (GenScript, NJ) (Table S4). Each peptide was synthesized with a 5-amino-acid sequence overlapping the previous peptide and prepared as stock solutions of 10 mM. Microplates containing immobilized streptavidin (2 µg/mL, Millipore) were blocked in 10% NFDM and incubated with 25 µM of each peptide in TBST overnight at RT. Binding of anti-VP2 MAbs 7B3- and 2D1-HRP (1 µg/mL) to each peptide was determined with a chemiluminescent substrate (Ultra ECL) using a Victor X3 luminometer and reported as CPS (*N* = 8).

### Statistical analysis

Graphical data are expressed as means ± SEM with no fewer than three independent samples. Absorbance data from the MTT were plotted on a log-linear scale, and the maximum cell viability was determined by mock infection, equivalent to SVA-infected NCI-H1299 CM dilutions > e + 12, and used to define the upper MTT assay threshold. The minimum cell viability was determined at the most concentrated viral dilutions (< e + 1) and defined the lower MTT assay threshold. The absorbance data plotted between the thresholds were analyzed by linear regression (*r*^2^ > 0.9) and used to calculate the dilution of virus that results in 50% cell viability (MTT50) as an equivalent to the TCID50 (tissue culture infectious dose 50) obtained by visual scoring of cellular cytopathic effect (CPE) after log dilution. Sandwich ELISA dose-response data were plotted with regression lines drawn using a dynamic four-parameter logistic curve-fit model (4PL) on log-linear scale. These data were used to calculate EC50 (half maximal concentration), hillslope, and limit of detection (LOD). The Shapiro–Wilk test was used to analyze data for normality, and Brown-Forsythe was used to analyze data for equal variance. Data that passed normality were analyzed by either Student’s *t*-test, when equal variance was assumed, or Welch’s *t*-test, when equal variance was not assumed. Pairwise comparison of samples to equivalent no-antigen or mock-infected controls was used to determine statistical significance and is reported as *P*-values, with probabilities >95% considered significant. Antibody binding data to synthetic VP2 peptides were analyzed by Kruskal-Wallis one-way analysis of variance on ranks (*P* ≤ 0.001) followed by Dunn’s method for multiple comparisons versus a negative control group (*P* ≤ 0.01). All plotted graphical data and statistical analysis were performed using SigmaPlot 15.0 software (GrafitiLLC, CA).

## RESULTS

Senecavirus A (SVA) was identified as a contaminant of cell culture (1), and pigs later shown to be the natural viral host (13, 25). Retrospective serosurveys suggest that the virus has been circulating in U.S. pigs since 1988 (26), and the virus has a widespread global prevalence (8). SVA is known to be oncolytic to a variety of cancers and will infect multiple human and porcine cell lines (27). In these studies, we use the well-characterized contemporary wild-type SVA SD15-26 strain (13, 28) for viral infection, replication, and evaluation.

**Fig 1:**
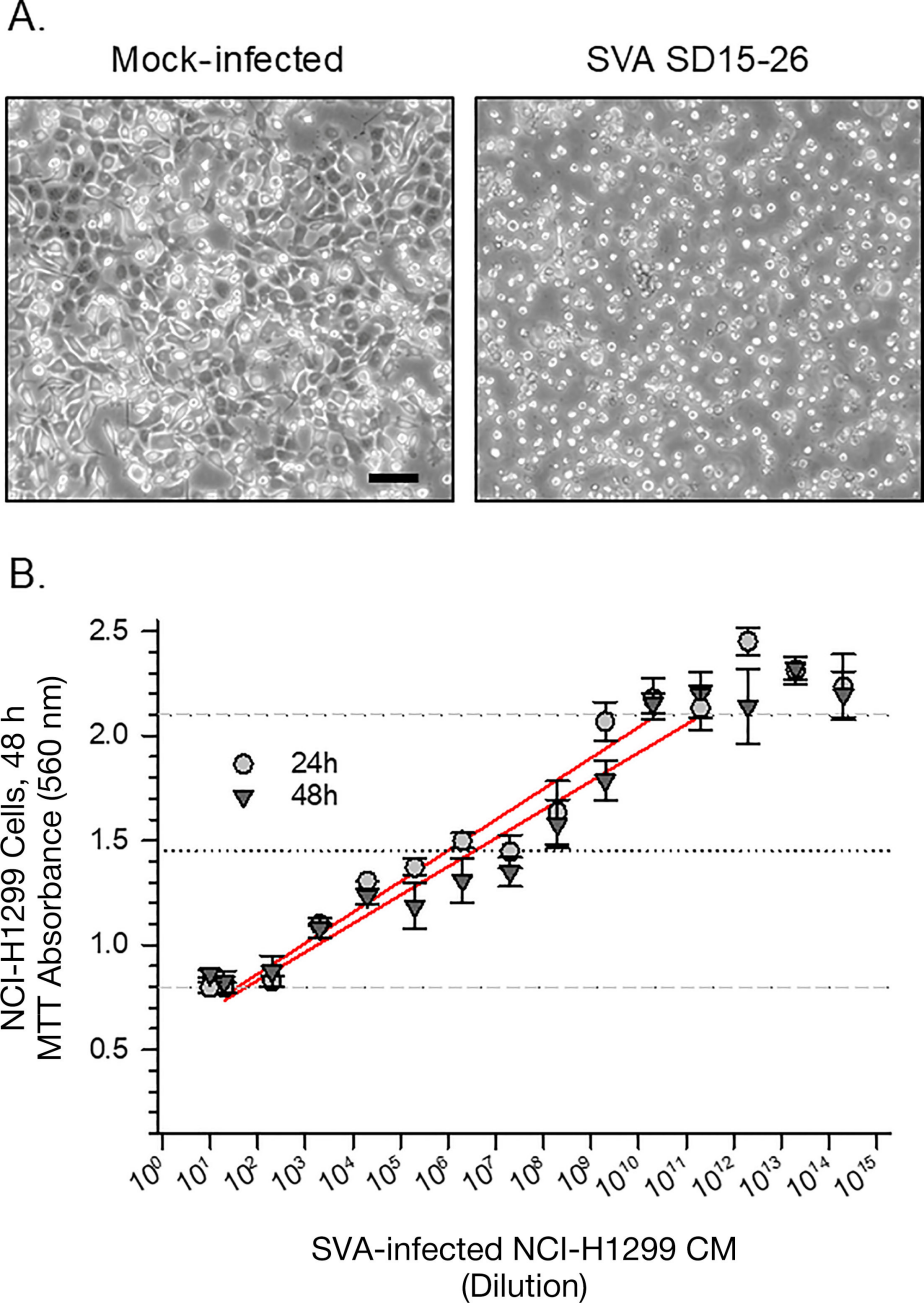
Phase-contrast photomicrographs of human lung NCI-H1299 epithelial cells (**A**) 48 h after mock (left panel) or SVA SD15-26 infection (right panel). Bar = 100 µm. An MTT assay was used to determine the dilution (MTT50) of SVA-infected samples that results in 50% cell viability of NCI-H1299 cells (**B**). SVA-infected cell-conditioned media (CM), collected at 24 h (circles) and 48 h (triangles), were log diluted and added to microplate wells (0.1 mL) containing 20K NCI-H1299 cells. After 48 h, 50 µL of MTT (5 mg/mL) was added to each well for 3 h, and the resulting metabolic formazan product from viable cells was dissolved and absorbance was measured at 560 nm (mean ± SEM). Maximal cell viability was defined as the absorbance threshold from mock-infected cells (top dashed line), and the lowest cell viability was defined as the absorbance threshold from the most concentrated viral samples (bottom dashed line). Linear regression was plotted (red lines) between the thresholds for SVA infected 24 h CM (*R*_2_ = 0.943) and 48 h CM (*R*_2_ = 0.935) with 50% viable cells (dotted line) reported as the dilution (MTT50) from the intersection of the regression lines. The MTT50/mL for the SVA-infected 24 h CM = 1.05e + 7 and 48 h CM = 3.40e + 7 equivalent to 7.35e + 5 and 2.38e + 6 viral particles, respectively.

**Fig 2:**
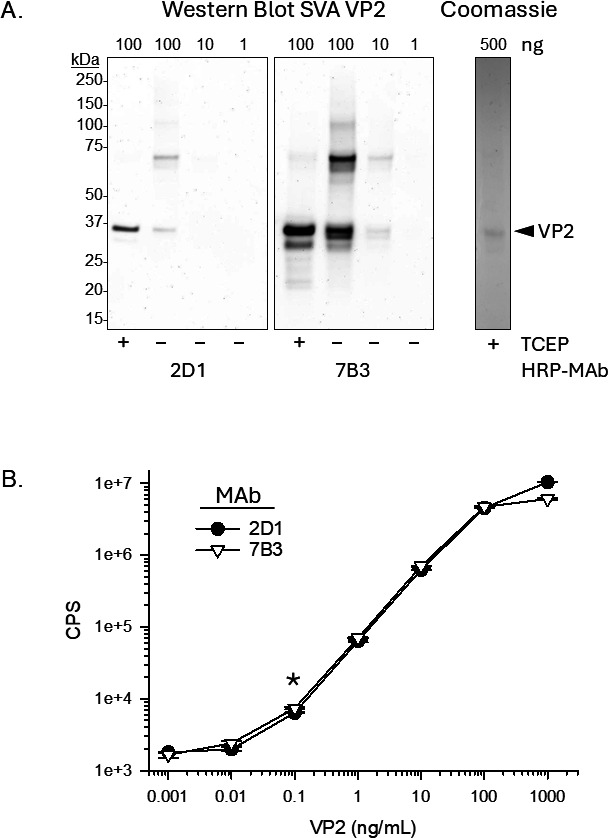
Western blot detection of the VP2 protein with 2D1 and 7B3 MAbs (**A**). Dilutions of purified recombinant VP2 protein were heat-denatured (−) and reduced in TCEP (+), separated by gel electrophoresis, and subjected to Western blotting with binding of anti-SVA VP2 MAbs 2D1- and 7B3-HRP conjugates visualized by chemiluminescent detection. Purified VP2 monomer migrates to an expected molecular weight of ~36 kDa (Coomassie stained gel; right panel). Detection of the VP2 protein with 2D1 and 7B3 MAb by direct ELISA (**B**). Log dilutions of the VP2 protein were immobilized on microplates (*N* = 4), and binding of the 2D1 (circles) and 7B3 (triangles) MAb-HRP conjugates was determined by luminometry. Data are plotted as mean ± SEM in CPS (counts per sec).

Figure 1A shows phase-contrast photomicrographs of the human NCI-H1299 non-small cell lung carcinoma cell line (ATCC CRL-5803) after 48 h SVA SD15-26 or mock infection. Uninfected cells exhibit a typical cuboid adherent epithelial appearance, with SVA infection resulting in cell rounding, detachment, and death. In Fig. 1B, we show the impact of log dilutions of infectious SVA on NCI-H1299 cells after 48 h using an MTT assay. This assay provides a measure of cell viability and proliferation by assessing the reduction of the yellow tetrazolium salt (MTT) to a purple formazan product by mitochondrial dehydrogenases. The formazan product can be measured spectrophotometrically (560 nm) and is directly proportional to the number of viable cells (29). The maximum MTT absorbance threshold was determined using uninfected cells (top dashed line), and the minimum threshold was defined by the most concentrated SVA viral dilutions (bottom dashed line), reflecting the persistent surviving cell population. Dilutions of SVA-infected cell-conditioned media (CM) collected after 24 h and 48 h were plotted between the assay thresholds and analyzed by first-order linear regression (*R*^2^ 24 h = 0.943 and *R*^2^ 48 h = 0.935). The dotted line represents the MTT absorbance midpoint with 50% cell viability (MTT50). The MTT50/mL is a measure of viral infectivity, reported as a dilution, with the CM collected 24 h = 1.05e + 7 and 48 h = 3.4e + 7 after SVA infection (30, 31). The viral particles in the samples are calculated from the MTT50/mL × 0.7 (a factor determined by Poisson distribution applied to viral infection in a fully permissive cell line with the probability of reaching 50% infection [32]), with SVA-infected 24 h CM = 7.35e + 5 and 48 h CM = 2.38e + 6 viral particles. The data show that the CM collected from SVA-infected cells after 48 h of viral exposure contains more infectious viral particles than the CM collected after 24 h. SVA has four structural proteins (VP1-4) that contribute to the formation of the protective viral capsid. The VP1-3 proteins are located at the capsid surface and are involved in host cell binding and entry (3). VP2 has been shown to elicit robust antibody responses and is used in serological-based assays (17, 19, 33, 34). SVA is the only member of the genus *Senecavirus*, and the VP2 protein is highly conserved among SVA variants (Table S1). Although other picornaviruses exhibit structural and organizational homologies, the primary structure of their VP2 proteins is divergent (Table S3A) and elicits no serological cross-reactivity (17). In this report, we generate and express a bacterial recombinant VP2 protein for mouse immunization and hybridoma screening for the selection of anti-VP2 monoclonal antibodies (MAbs). A cohort of clonal hybridoma cells, all producing anti-VP2 MAbs, was evaluated, with only the 2D1 and 7B3 MAb binding virus (Table S2). In Fig. 2A, we show the binding of the 2D1 and 7B3 MAb to recombinant VP2 by Western blot. These MAbs bind both denatured and reduced VP2, with binding of the 7B3 > 1 log more sensitive (10 ng) than the 2D1 MAb. A log dilution series of the VP2 protein was immobilized in microplates, and the binding of both 2D1 and 7B3 MAb was evaluated by direct ELISA (Fig. 2B). The VP2 limit of detection (LOD) for both MAbs is 100 pg/mL (*P* ≤ 0.001), with linear binding over 4 logs (*R*^2^ 2D1 = 0.976 and *R*^2^ 7B3 = 0.948).

**Fig 3:**
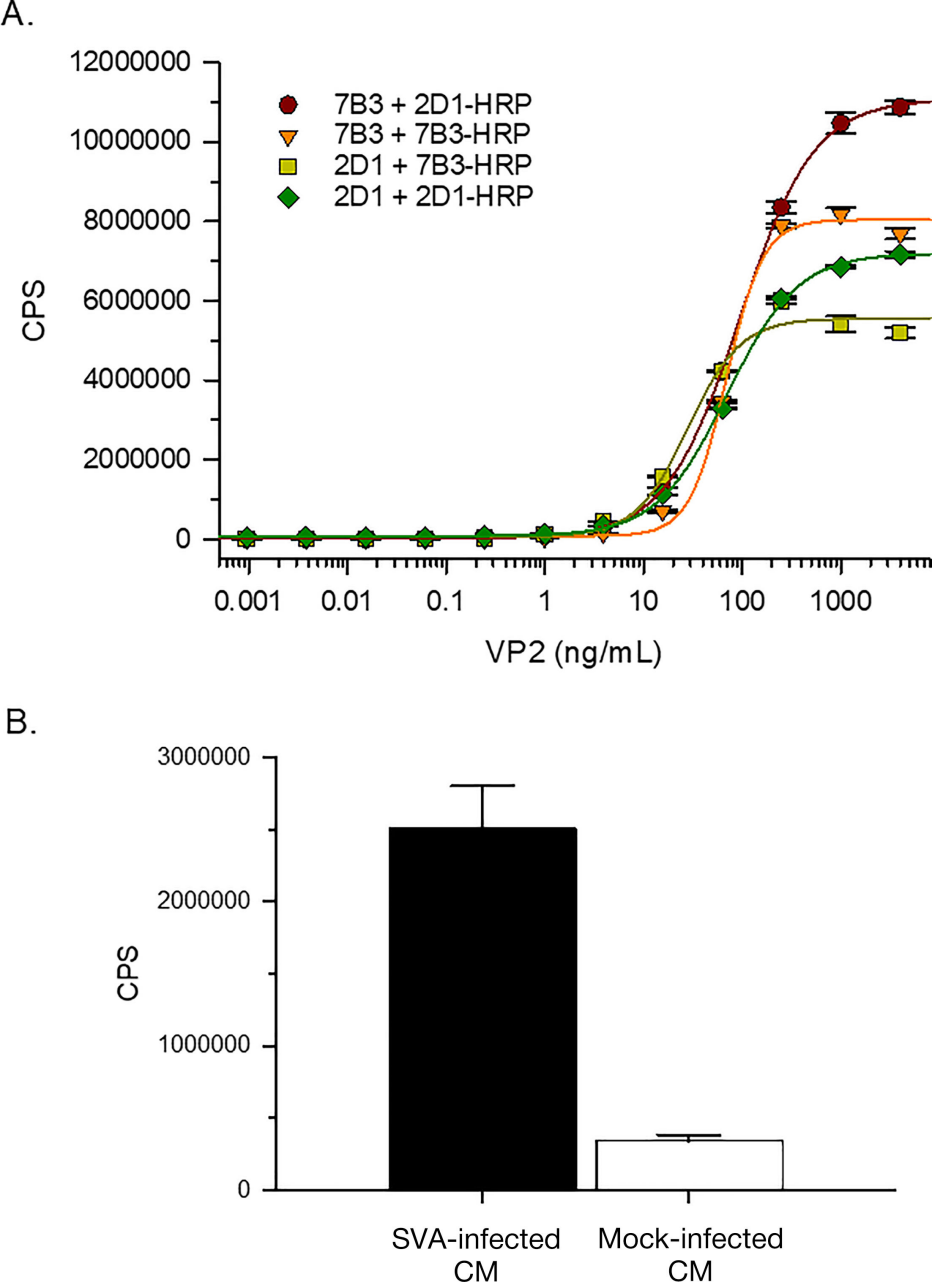
Detection of recombinant VP2 protein and SVA SD15-26 virus by sandwich ELISA. Binding of MAb pair combinations (2D1 and 7B3) to log dilutions of VP2 was determined by chemiluminescent sELISA and data plotted as mean ± SEM CPS (*N* = 3). Data were subjected to dynamic four-parameter logistic curve fitting (4PL) and non-linear regression lines graphed (**A**). Microplate-immobilized 7B3 MAb was paired with the 2D1-HRP MAb reporter and used to detect SVA SD15-26 virus from infected or mock-infected NCI-H1299 cell-conditioned media (CM) by sandwich ELISA (**B**). CPS, counts per sec.

The sELISA is an important tool used for the detection and quantitation of a target antigen from a wide range of substrates (35, 36). To facilitate sELISA development, we selected MAbs with VP2 capture capability as part of our screening strategy. Only a small subset of antibodies will exhibit antigen-binding properties suitable for an sELISA format. In Fig. 3A, we report the detection of VP2 from a log dilution series with pairwise combinations of the 2D1 and 7B3 MAbs by sELISA. In this format, one MAb is immobilized on the microplate for VP2 capture while another, functionalized with an HRP reporter, is used for the detection of the captured VP2 by either luminometry after the addition of a chemiluminescent substrate or absorbance at 560 nm after the addition of a blue colorimetric TMB substrate (Fig. S2). These two MAb combinations, including self-pairing, all function in the sELISA for the detection of VP2. Table 1 reports the LOD (*P* ≤ 0.001), EC50 (ng/mL), and hillslope from curve-fit data (4PL) for each sELISA pair combination. Although the orientation of the 7B3 and 2D1 MAbs, as either the capture or detector, had minimal impact on assay performance (LOD = 61 pg/mL), we determined the 7B3 and 2D1-HRP MAb pair orientation to be optimal based on curve-fit data. The detection of wild-type SVA SD15-26 by sELISA from NCI-H1299 cell media (CM) after 3 days is shown in Fig. 3B (luminometry) and Fig. S2 (absorbance) using the 7B3 and 2D1-HRP MAb pair.Figure S1Ashows detection of two alternate SVA strains (SVV-001/2002 and US-HI/NADC40/2012) from CM by the sELISA. The detection of SVA by sELISA is significant (*P* ≤ 0.001), with the chemiluminescent signal (CPS) >8× relative to uninfected control (mock CM). The use of the recombinant VP2 sELISA as a standard curve predicts a VP2 concentration of 3 ng/mL in the SVA-infected CM. These data demonstrate that both the 7B3 and 2D1 MAbs bind available VP2 epitopes for the detection of intact SVA virus.

**TABLE 1:** EC50 and hillslopes for each VP2 sELISA standard curve were determined for MAb pair combinations by dynamic four-parameter logistic curve fit^*a*^

Immobilized capture MAb	HRP-reporter detection MAb	EC50 (ng/mL)	Hillslope	LOD (pg/mL)
7B3	2D1	94.6	1.15	61
7B3	7B3	69.6	2.32	61
2D1	7B3	28.3	1.63	61
2D1	2D1	70.5	1.22	244

^*a*^ The limit of detection (LOD) was determined for each sELISA by comparing the mean chemiluminescent detection of VP2 concentrations to no-antigen control using Student’s *t*-test (*N *= 4). Statistical significance for each LOD was *P* ≤ 0.001.

**Fig 4:**
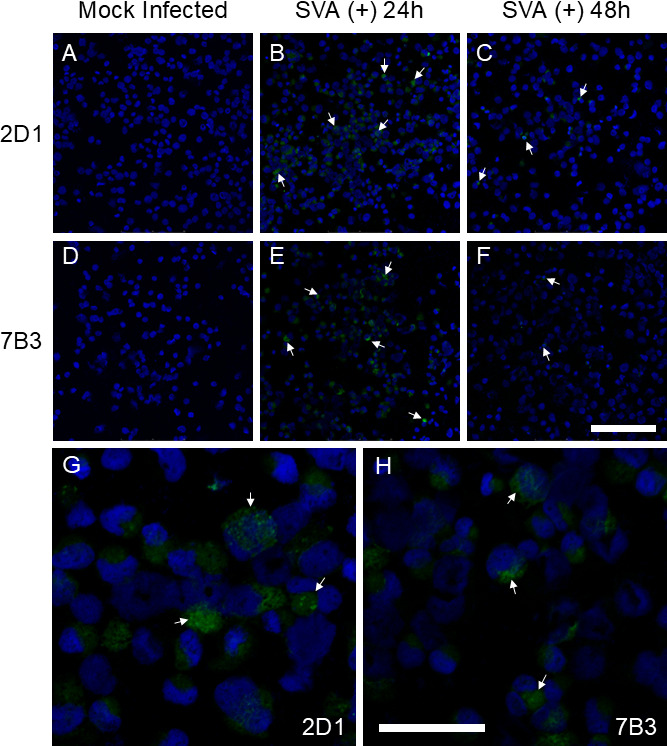
Immunofluorescent detection of SVA SD15-26 in NCI-H1299 infected cells. Photomicrographs of green immunofluorescent binding of 2D1 and 7B3 MAbs to SVA SD15-26-infected NCI-H199 cells after 24 h and 48 h. (**A–C**) 2D1 FITC. (**D–F**) 7B3 FITC. (**A–F**) Bar = 250 µm. (**G**) 2D1 FITC after 24 h. (**H**) 7B3 FITC after 24 h. (**G and H**) Bar = 75 µm. Arrows point to green immunofluorescent detection of SVA. Mock-infected cells = negative control. Cell nuclei are counterstained with blue DAPI.

Immunofluorescent photomicrographs of 2D1 and 7B3 MAb binding SVA-infected NCI-H1299 cells are shown in Fig. 4. SVA binding is detected as a green fluorescence (FITC) with individual cell nuclei resolved in blue (DAPI). No green fluorescence was observed in uninfected mock control cells, but SVA detection with both MAbs is observed in cells at both 24 h and 48 h post-infection. Both MAbs reported more SVA-positive cells at the 24 h time point compared to 48 h post-infection. This data provides further evidence that these MAbs bind SVA at available VP2 epitopes on the intact viral capsid.

**Fig 5:**
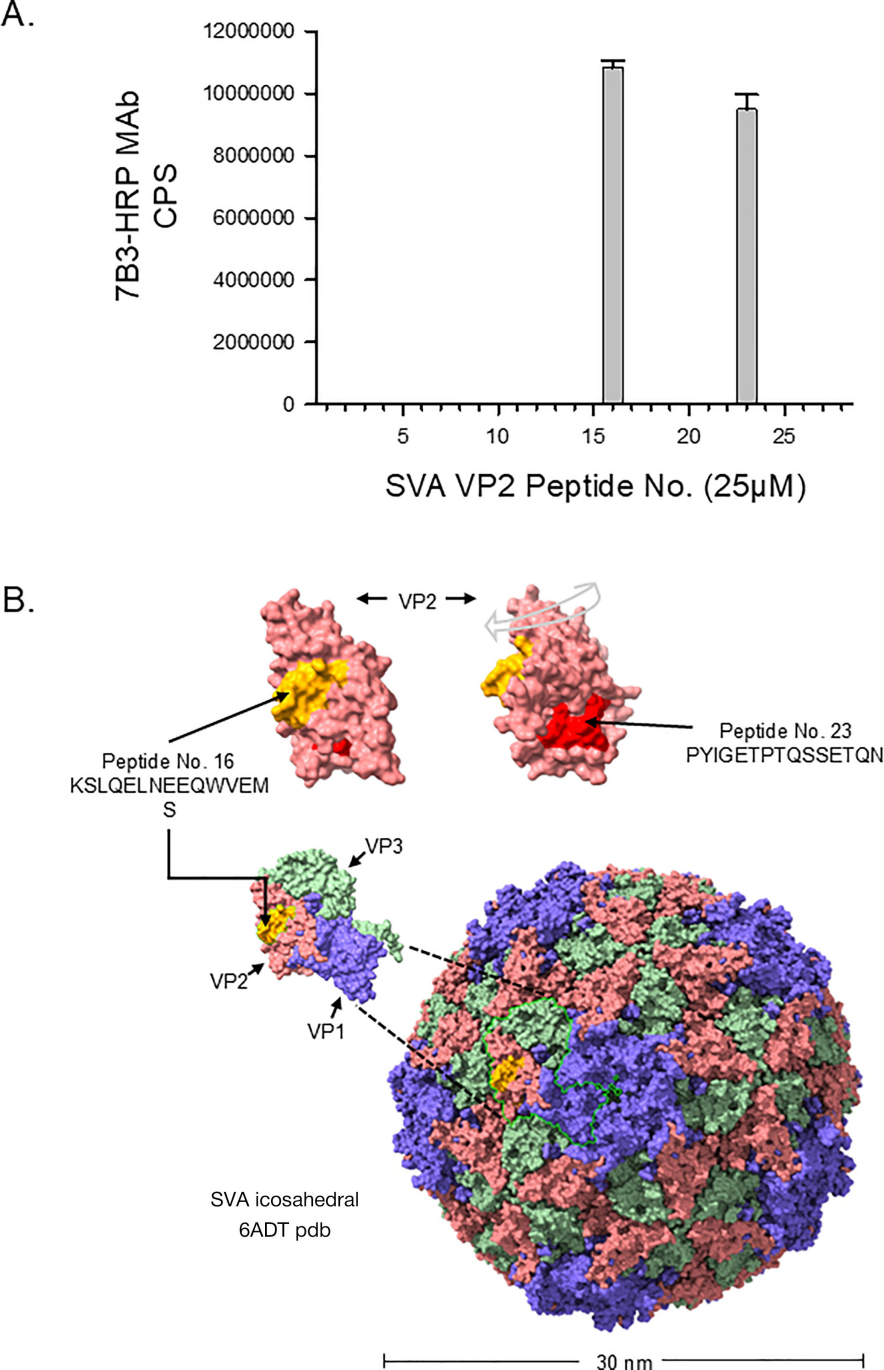
Anti-VP2 MAb binding epitopes. A series of VP2 peptides (*N* = 28), each 15-amino acid long with a 5-amino acid overlap, were immobilized, and chemiluminescent binding of the 7B3-HRP MAb was determined by ELISA (**A**). 7B3 MAb peptide binding data is plotted as mean ± SEM CPS (*N* = 8), with binding to peptides 16 and 23 considered significant (*P* < 0.001). No significant peptide binding was reported for the 2D1 MAb (data not shown). A putative 7B3 MAb binding epitope was mapped to the 3D VP2 protein and SVA icosahedral structures (**B**). Peptide-16 (yellow) and peptide-23 (red) are color-coded on a space-filled VP2 monomer (pink). The interaction of VP2 (pink) with VP1 (purple) and VP3 (green) is illustrated and shown in the composite SVA icosahedral structure (6ADT). The location of amino acid residues from peptide-16 (yellow) to the external face of VP2 at the surface of the SVA icosahedral suggests their strong contribution to the 7B3 MAb binding epitope.

To further elucidate the MAb binding epitopes, we constructed a linear series of 28 biotinylated peptides (15 aa with 5 aa overlap) that correspond to the VP2 primary structure (Table S4). Microplate-immobilized streptavidin was used to capture the N-terminal biotinylated peptides, and MAb binding was determined by dELISA. No significant binding was reported to any peptide using the 2D1 MAb (data not shown). In Fig. 5A, we report statistically significant 7B3 MAb binding to peptide-16 and peptide-23 (*P* < 0.001). These peptides are mapped to the space-filled 3D structure of VP2 (37) with their relative location shown in Fig. 5B as part of the composite SVA capsid icosahedron (38) (pdb6ADT). VP2 is illustrated in pink, with peptide-16 colored yellow (residues 150–164) and peptide-23 (residues 220–234) colored red. VP structural proteins are color-coded (VP1 = purple, VP2 = pink, VP3 = green, and VP4 = tan) with the inner orientation of the VP4 protein not visible. The complete capsid is made of 60 copies of each VP protein, and we highlight one unit (VP1-4) in green outline to show the external orientation of peptide-16 VP2 residues (yellow) to the viral capsid surface. The location of peptide-16 residues of VP2 at the capsid surface strongly suggests that these residues contribute to the 7B3 MAb binding epitope.

## DISCUSSION

SVA infections are clinically indistinguishable from other serious transboundary viral vesicular diseases, and the widespread prevalence of SVA in pigs has resulted in increased FAD investigations by the USDA (4, 9, 11). These investigations can be time-consuming, expensive, and disruptive to supply chains (10). A rapid, inexpensive, and easy-to-perform pen-side test for SVA detection in clinically suspect animals would be a useful tool to facilitate FAD investigations and reduce the economic burden of supply chain disruptions. In this manuscript, we describe the generation of MAbs against the SVA VP2 protein and report the development of a sensitive sELISA for the direct detection of the SVA virus.

The SVA VP2 protein has been used effectively in serological assays as an antigen for the detection of circulating anti-VP2 antibodies, suggesting that some VP2 epitopes are accessible immunogenic targets for viral defense (19, 33, 34). We generated a cohort of anti-VP2 MAb that functions to capture and detect recombinant SVA VP2 protein by sELISA, but only two were capable of native virus detection. This is likely a function of multiple factors that may include artifacts of recombinant VP2 production, the availability of the VP2 binding epitope at the viral surface, or the availability of the VP2 epitope after capsid assembly. As demonstrated in Fig. 2A, recombinant VP2 protein migrates as a dimer (~72 kDa) that is sensitive to chemical reduction (~36 kDa), suggesting formation of external disulfide bonds. This VP2 dimer probably does not occur during the coordinated process of viral replication and capsid assembly. Indeed, Fig. 3A shows that the anti-VP2 MAbs will self-pair in sELISA, demonstrating that two available VP2 epitopes also occur in solution. This data re-enforces the need for careful screening and selection strategies when generating MAbs for applications targeting complex structural assemblies using a single recombinant protein. Self-pairing of the 7B3 and 2D1 MAbs in sELISA for the native virus is not a function of VP2 dimerization but rather redundancy of the VP2 in the viral capsid. In Fig. 4, we show the binding of these anti-VP2 MAbs to formalin-fixed cells at 24 h and 48 h post-infection. These data visualize virus particles with green immunofluorescence and suggest that more SVA is cell-associated at the 24 h versus 48 h time post-infection. This difference might reflect time differences in the viral life cycle, such as pre-protein expression of VP0 prior to cleavage into VP2 and VP4, with non-cell-associated virus being undetectable by this technique. These anti-VP2 MAbs are both capable of affinity isolation of the virus following amine immobilization of MAb to agarose beads (data not shown). In addition, these MAbs will function in a serum ELISA useful for the serological evaluation of animals that have been infected with SVA by competitive inhibition of MAb binding by VP2 antibodies in serum (Fig. S3).

The 7B3 and 2D1 MAb binding by Western blot of heat-denatured VP2 protein suggests both can bind linear protein epitopes (Fig. 2A). This binding is independent of disulfide bonds, as indicated by the protein band shift and increased intensity of the 36 kDa VP2 monomer after TCEP reduction. The 7B3 MAb binding to VP2 is of higher intensity compared to the 2D1 MAb, resulting in a log-order increase in detection sensitivity. Unlike the Western blot, we observe no difference in VP2 binding by dELISA with either the 7B3 or 2D1 MAbs (Fig. 2B). This suggests that the binding of the 2D1 MAb is negatively impacted by protein unfolding following heat denaturation. This evidence is further supported by the failure of the 2D1 MAb to bind any of the 28 × 15 aa linear VP2 peptides in solution, suggesting that the 2D1 MAb binding epitope differs from the 7B3 MAb and prefers a more native conformation of the VP2 protein structure. The 7B3 MAb bound two linear VP2 peptides (Fig. 5A), with amino acids from peptide-16 (KSLQELNEEQWVEMS) mapping to the surface of the viral capsid (Fig. 5B). No significant binding of the 7B3 MAb was observed with either peptide-15 or peptide-17, suggesting that the core canonical aa sequence LNEEQ plays an important role in the 7B3 binding epitope. Significant binding is also observed to peptide-23 (PYIGETPTQSSETQN) with the core aa TPTQS residues, which are, by conformation, proximal to peptide-16 and participate in the 7B3 MAb binding epitope. These VP2 amino acid residues (150–164 and 220–234) do not appear to involve those implicated in SVA binding to the anthrax toxin receptor 1 (VP2: 142–151 and 172–200) used in viral cell entry (21, 38). This 7B3 MAb binding epitope is present in the SVA VP2 protein of both early (SVV-001-2002 and US-HI-2012) and contemporary (SD15-26) SVA strains (Fig. 3B; Fig. S1A and Fig. S1B ) as well as in all the SVA VP2 proteins compared by multiple sequence alignment (Table S1). These data predict broad binding of the 7B3 MAb to all existing SVA strains with no cross-reactivity to other picornaviruses associated with vesicular diseases, given the absence of the MAb binding epitope (Table S3A).

The importance of these MAbs and their use in immunoassay applications is their ability to detect the SVA virus, not VP2. Toward this aim, we show detection of multiple SVA strains (Fig. S2) along with the highly pathogenic contemporary SVA SD15-26 strain (Fig. 3B), which is associated with the post-2015 surge in SVA outbreaks. SVA-infected animals develop short-term viremia with shedding from oral or nasal secretions and feces for up to 28 days (13), with detectable virus found in both oral fluids and vesicular lesions (9, 12, 16). Future research will validate immunoassay performance across animal matrices and benchmark against RT-qPCR to define clinical sensitivity and specificity. Together, these findings demonstrate that the VP2-targeted MAbs 7B3 and 2D1 recognize conserved, surface-accessible epitopes on the SVA capsid and are uniquely capable of detecting the native virus across multiple assay platforms. By pairing robust antigen recognition with broad strain coverage, these immunoassays have strong potential to enhance diagnostic capacity, support differential diagnosis during FAD investigations, and reduce the economic and operational burden imposed by SVA-associated disease outbreaks.

## Data Availability

All data supporting the findings of this study are available from the corresponding author upon reasonable request.

## SUPPLEMENTAL MATERIAL

Fig. S1A jvi.00571-26-s0001.jpg Detection of SVA strains by sELISA.

Fig. S1B jvi.00571-26-s0002.jpg Multiple sequence alignment of VP2 from tested SVA strains.

Fig. S2 jvi.00571-26-s0003.jpg Colorimetric detection of SVA and VP2 by sELISA.

Fig. S3 jvi.00571-26-s0004.jpg Serum inhibition ELISA.

Table S1 jvi.00571-26-s0005.docx Multiple sequence alignment of SVA VP2 proteins.

Table S2 jvi.00571-26-s0006.docx Immunoassay binding properties of VP2 antibodies.

Table S3A jvi.00571-26-s0007.docx Multiple sequence alignment of picornavirus VP2 proteins.

Table S3B jvi.00571-26-s0008.docx Pairwise alignment of VP2 protein from SVA and FMDV.

Table S4 jvi.00571-26-s0009.docx SVA VP2 peptides used for epitope mapping.

ASM does not own the copyrights to Supplemental Material that may be linked to, or accessed through, an article. The authors have granted ASM a non-exclusive, world-wide license to publish the Supplemental Material files. Please contact the corresponding author directly for reuse.
