# Supplementary figures and images for: VP2-targeted sandwich ELISA (sELISA) enables direct detection of Senecavirus A (SVA)
Source: J Virol. 2026 May 12;100(6):e00571-26. doi: 10.1128/jvi.00571-26 (PMC13289164; doi:10.1128/jvi.00571-26)

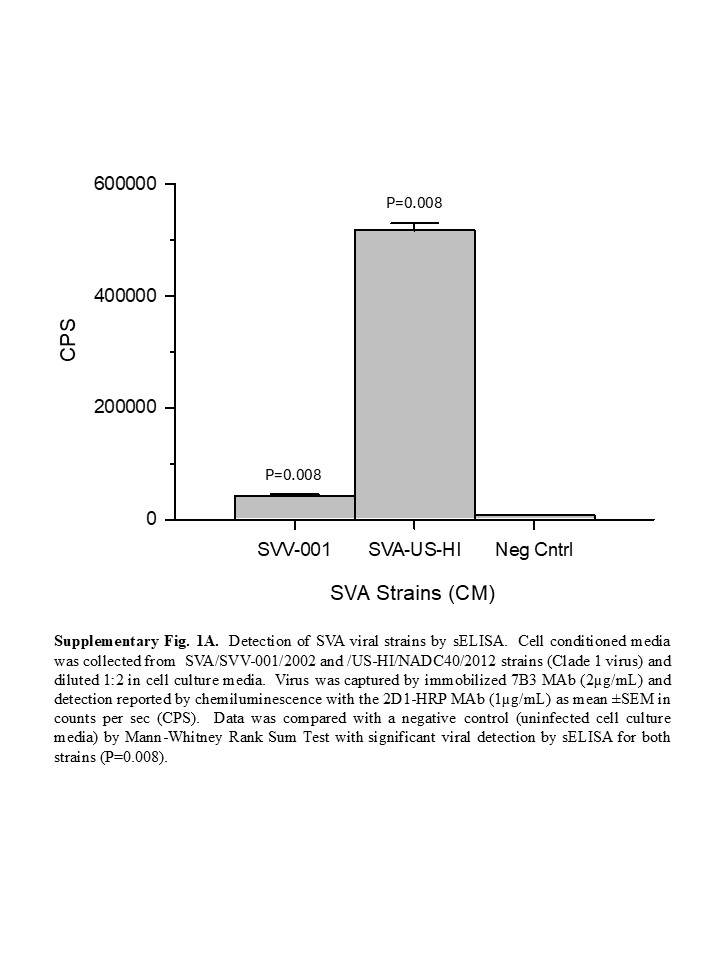

Supplement: Fig. S1A — Detection of SVA strains by sELISA. [file jvi.00571-26-s0001.jpg]

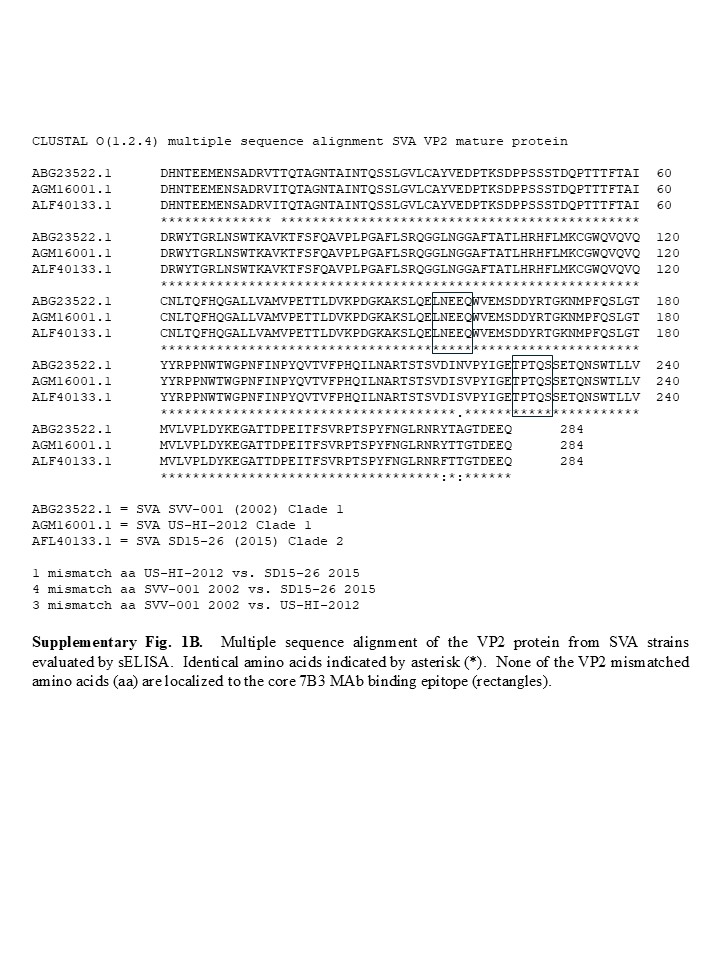

Supplement: Fig. S1B — Multiple sequence alignment of VP2 from tested SVA strains. [file jvi.00571-26-s0002.jpg]

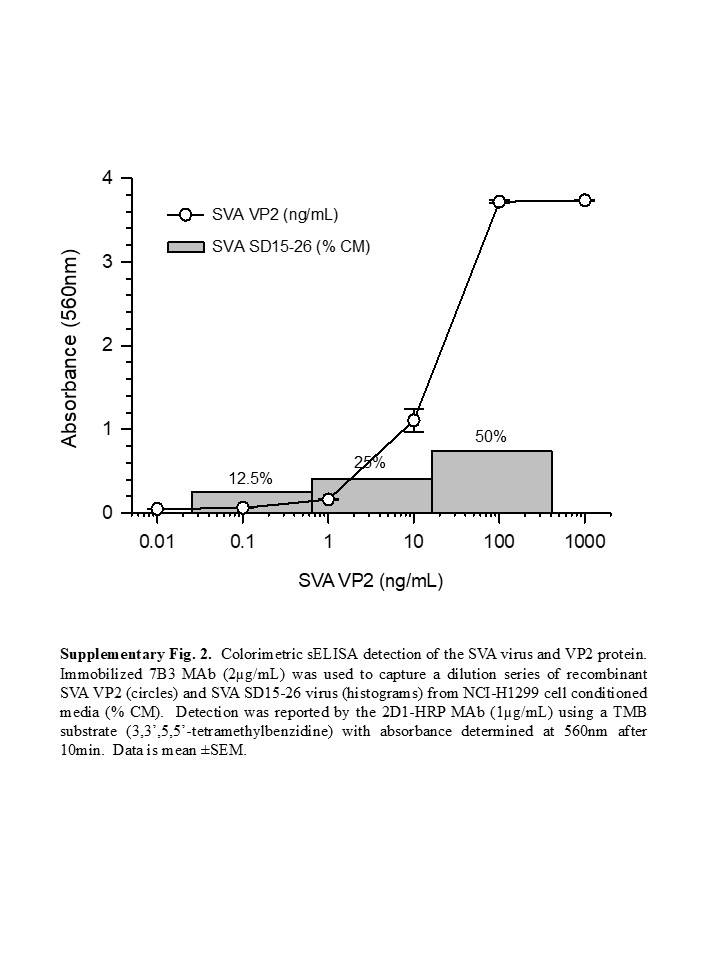

Supplement: Fig. S2 — Colorimetric detection of SVA and VP2 by sELISA. [file jvi.00571-26-s0003.jpg]

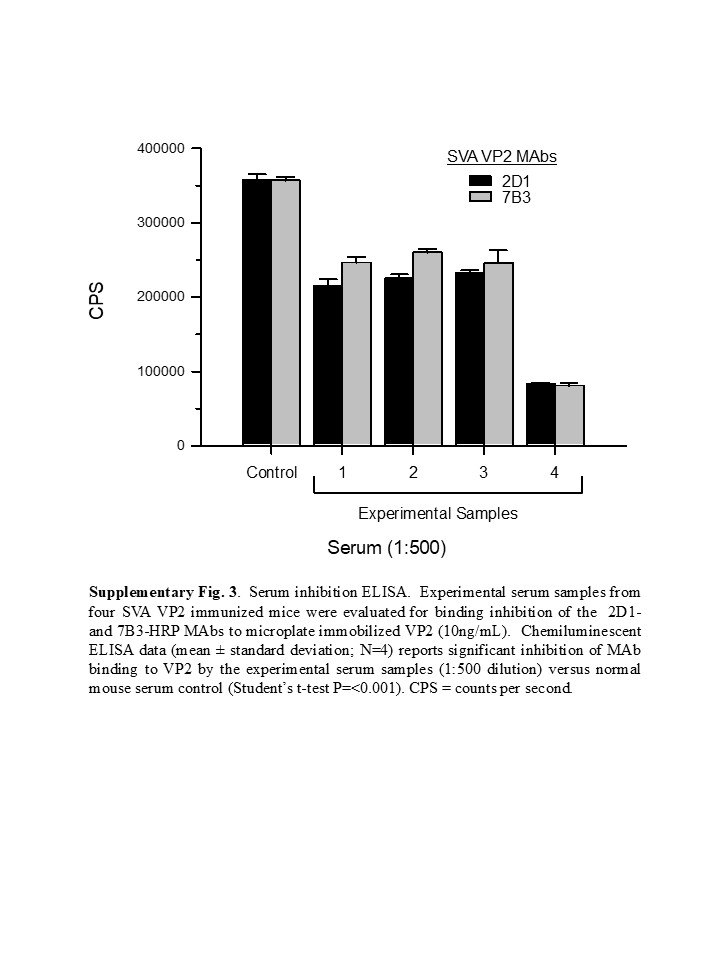

Supplement: Fig. S3 — Serum inhibition ELISA. [file jvi.00571-26-s0004.jpg]
