# Supplementary material for: VP2-targeted sandwich ELISA (sELISA) enables direct detection of Senecavirus A (SVA)
Source: J Virol. 2026 May 12;100(6):e00571-26. doi: 10.1128/jvi.00571-26 (PMC13289164; doi:10.1128/jvi.00571-26)
Supplement: Table S1 — Multiple sequence alignment of SVA VP2 proteins. [file jvi.00571-26-s0005.docx]

CLUSTAL O(1.2.4) multiple sequence alignment SVA VP2 protein

QWW20846.1 -DHNTEEMENSADRVTTQTAGNTAINTQSSLGVLCAYVEDPTKSDPPSSSTDQPTTTFTA 59

WBL45177.1 -DHNTEEMENSADRVITQTAGNTAINTQSSLGVLCAYVEDPTKSDPPSSSTDQPTTTFTA 59

XCJ77342.1 -DHNTEEMENSADRVITQTAGNTAINTQSSLGVLCAYVEDPTKSDPPSSSTDQPTTTFTA 59

QWW20845.1 -DHNTEEMENSADRVITQTAGNTAINTQSSLGVLCAYVEDPTKSDPPSSSTDQPTTTFTA 59

XBN89312.1 -DHNTEEMENSADRVITQTAGNTAINTQSSLGVLCAYVEDPTKSDPPSSSTDQPTTTFTA 59

XCJ77331.1 -DHNTEEMENSADRVITQTAGNTAINTQSSLGVLCAYVEDPTKSDPPSSSTDQPTTTFTA 59

XCJ77335.1 -DHNTEEMENSADRVITQTAGNTAINTQSSLGVLCAYVEDPTKSDPPSSSTDQPTTTFTA 59

XBN89309.1 -DHNTEEMENSADRVITQTAGNTAINTQSSLGVLCAYVEDPTKSDPPSSSTDQPTTTFTA 59

XBN89307.1 -DHNTEEMENSADRVITQTAGNTAINTQSSLGVLCAYVEDPTKSDPPSSSTDQPTTTFTA 59

XBN89295.1 -DHNTEEMENSADRVITQTAGNTAINTQSSLGVLCAYVEDPTKSDPPSSSTDQPTTTFTA 59

XBN89292.1 -DHNTEEMENSADRVITQTAGNTAINTQSSLGVLCAYVEDPTKSDPPSSSTDQPTTTFTA 59

XBN89293.1 -DHNTEEMENSADRVITQTAGNTAINTQSSLGVLCAYVEDPTKSDPPSSSTDQPTTTFTA 59

XBN89294.1 -DHNTEEMENSADRVITQTAGNTAINTQSSLGVLCAYVEDPTKSDPPSSSTDQPTTTFTA 59

XBN89296.1 -DHNTEEMENSADRVITQTAGNTAINTQSSLGVLCAYVEDPTKSDPPSSSTDQPTTTFTA 59

XBN89297.1 -DHNTEEMENSADRVITQTAGNTAINTQSSLGVLCAYVEDPTKSDPPSSSTDQPTTTFTA 59

XBN89301.1 -DHNTEEMENSADRVITQTAGNTAINTQSSLGVLCAYVEDPTKSDPPSSSTDQPTTTFTA 59

XBN89302.1 -DHNTEEMENSADRVITQTAGNTAINTQSSLGVLCAYVEDPTKSDPPSSSTDQPTTTFTA 59

XBN89310.1 -DHNTEEMENSADRVITQTAGNTAINTQSSLGVLCAYVEDPTKSDPPSSSTDQPTTTFTA 59

XBN89313.1 -DHNTEEMENSADRVITQTAGNTAINTQSSLGVLCAYVEDPTKSDPPSSSTDQPTTTFTA 59

XBN89315.1 -DHNTEEMENSADRVITQTAGNTAINTQSSLGVLCAYVEDPTKSDPPSSSTDQPTTTFTA 59

XBN89316.1 -DHNTEEMENSADRVITQTAGNTAINTQSSLGVLCAYVEDPTKSDPPSSSTDQPTTTFTA 59

XBN89317.1 -DHNTEEMENSADRVITQTAGNTAINTQSSLGVLCAYVEDPTKSDPPSSSTDQPTTTFTA 59

XBN89318.1 -DHNTEEMENSADRVITQTAGNTAINTQSSLGVLCAYVEDPTKSDPPSSSTDQPTTTFTA 59

XBN89319.1 -DHNTEEMENSADRVITQTAGNTAINTQSSLGVLCAYVEDPTKSDPPSSSTDQPTTTFTA 59

XCJ77326.1 -DHNTEEMENSADRVITQTAGNTAINTQSSLGVLCAYVEDPTKSDPPSSSTDQPTTTFTA 59

XCJ77327.1 -DHNTEEMENSADRVITQTAGNTAINTQSSLGVLCAYVEDPTKSDPPSSSTDQPTTTFTA 59

XCJ77332.1 -DHNTEEMENSADRVITQTAGNTAINTQSSLGVLCAYVEDPTKSDPPSSSTDQPTTTFTA 59

XCJ77333.1 -DHNTEEMENSADRVITQTAGNTAINTQSSLGVLCAYVEDPTKSDPPSSSTDQPTTTFTA 59

XCJ77334.1 -DHNTEEMENSADRVITQTAGNTAINTQSSLGVLCAYVEDPTKSDPPSSSTDQPTTTFTA 59

QWW20847.1 -DHNTEEMENSADRVITQTAGNTAINTQSSLGVLCAYVEDPTKSDPPSSSTDQPTTTFTA 59

YAJ45130.1 -DHNTEEMENSADRVITQTAGNTAINTQSSLGVLCAYVEDPTKSDPPSSSTDQPTTTFTA 60

YAJ45131.1 -DHNTEEMENSADRVITQTAGNTAINTQSSLGVLCAYVEDPTKSDPPSSSTDQPTTTFTA 59

XBN89298.1 -DHNTEEMENSADRVITQTAGNTAINTQSSLGVLCAYVEDPTKSDPPSSSTDQPTTTFTA 59

XBN89299.1 -DHNTEEMENSADRVITQTAGNTAINTQSSLGVLCAYVEDPTKSDPPSSSTDQPTTTFTA 59

XBN89300.1 -DHNTEEMENSADRVITQTAGNTAINTQSSLGVLCAYVEDPTKSDPPSSSTDQPTTTFTA 59

XBN89303.1 -DHNTEEMENSADRVITQTAGNTAINTQSSLGVLCAYVEDPTKSDPPSSSTDQPTTTFTA 59

XBN89304.1 -DHNTEEMENSADRVITQTAGNTAINTQSSLGVLCAYVEDPTKSDPPSSSTDQPTTTFTA 59

XBN89305.1 -DHNTEEMENSADRVITQTAGNTAINTQSSLGVLCAYVEDPTKSDPPSSSTDQPTTTFTA 59

XBN89306.1 -DHNTEEMENSADRVITQTAGNTAINTQSSLGVLCAYVEDPTKSDPPSSSTDQPTTTFTA 59

XBN89308.1 -DHNTEEMENSADRVITQTAGNTAINTQSSLGVLCAYVEDPTKSDPPSSSTDQPTTTFTA 59

XBN89311.1 -DHNTEEMENSADRVITQTAGNTAINTQSSLGVLCAYVEDPTKSDPPSSSTDQPTTTFTA 59

XBN89314.1 -DHNTEEMENSADRVITQTAGNTAINTQSSLGVLCAYVEDPTKSDPPSSSTDQPTTTFTA 59

XBN89320.1 -DHNTEEMENSADRVITQTAGNTAINTQSSLGVLCAYVEDPTKSDPPSSSTDQPTTTFTA 59

XCJ77328.1 -DHNTEEMENSADRVITQTAGNTAINTQSSLGVLCAYVEDPTKSDPPSSSTDQPTTTFTA 59

XCJ77329.1 -DHNTEEMENSADRVITQTAGNTAINTQSSLGVLCAYVEDPTKSDPPSSSTDQPTTTFTA 59

XCJ77330.1 -DHNTEEMENSADRVITQTAGNTAINTQSSLGVLCAYVEDPTKSDPPSSSTDQPTTTFTA 59

XCJ77336.1 -DHNTEEMENSADRVITQTAGNTAINTQSSLGVLCAYVEDPTKSDPPSSSTDQPTTTFTA 59

XCJ77337.1 -DHNTEEMENSADRVITQTAGNTAINTQSSLGVLCAYVEDPTKSDPPSSSTDQPTTTFTA 59

XCJ77338.1 -DHNTEEMENSADRVITQTAGNTAINTQSSLGVLCAYVEDPTKSDPPSSSTDQPTTTFTA 59

XCJ77339.1 -DHNTEEMENSADRVITQTAGNTAINTQSSLGVLCAYVEDPTKSDPPSSSTDQPTTTFTA 59

XCJ77340.1 -DHNTEEMENSADRVITQTAGNTAINTQSSLGVLCAYVEDPTKSDPPSSSTDQPTTTFTA 59

XCJ77341.1 -DHNTEEMENSADRVITQTAGNTAINTQSSLGVLCAYVEDPTKSDPPSSSTDQPTTTFTA 59

XCJ77343.1 -DHNTEEMENSADRVITQTAGNTAINTQSSLGVLCAYVEDPTKSDPPSSSTDQPTTTFTA 59

UEW62688.1 -DHNTEEMENSADRVITQTAGNTAINTQSSLGVLCAYVEDPTKSDPPSSSTDQPTTTFTA 59

UEW62689.1 -DHNTEEMENSADRVITQTAGNTAINTQSSLGVLCAYVEDPTKSDPPSSSTDQPTTTFTA 59

UEW62690.1 -DHNTEEMENSADRVITQTAGNTAINTQSSLGVLCAYVEDPTKSDPPSSSTDQPTTTFTA 59

UEW62691.1 -DHNTEEMENSADRVITQTAGNTAINTQSSLGVLCAYVEDPTKSDPPSSSTDQPTTTFTA 59

UEW62692.1 -DHNTEEMENSADRVITQTAGNTAINTQSSLGVLCAYVEDPTKSDPPSSSTDQPTTTFTA 59

UEW62693.1 -DHNTEEMENSADRVITQTAGNTAINTQSSLGVLCAYVEDPTKSDPPSSSTDQPTTTFTA 59

QWW20848.1 -DHNTEEMENSADRVITQTAGNTAINTQSSLGVLCAYVEDPTKSDPPSSSTDQPTTTFTA 59

QWW20849.1 -DHNTEEMENSADRVITQTAGNTAINTQSSLGVLCAYVEDPTKSDPPSSSTDQPTTTFTA 59

QWW20850.1 -DHNTEEMENSADRVITQTAGNTAINTQSSLGVLCAYVEDPTKSDPPSSSTDQPTTTFTA 59

************** ********************************************

QWW20846.1 IDRWYTGRLNSWTKAVKTFSFQAVPLPGAFLSRQGGLNGGAFTATLHRHFLMKCGWQVQV 119

WBL45177.1 IDRWYTGRLNSWTKAVKTFSFQAVLLPGAFLSRQGGLNGGAFTATLHRHFLMKCGWQVQV 119

XCJ77342.1 IDRWYTGRLNSWTKAVKTFSFQAVPLPGAFLSRQGGLNGGAFTATLHRHFLMKCGWQVQV 119

QWW20845.1 IDRWYTGRLNSWTKAVKTFSFQAVPLPGAFLSRQGGLNGGAFTATLHRHFLMKCGWQVQV 119

XBN89312.1 IDRWYTGRLNSWTKAVKTFSFQAVPLPGAFLSRQGGLNGGAFTATLHRHFLMKCGWQVQV 119

XCJ77331.1 IDRWYTGRLNSWTKAVKTFSFQAVPLPGAFLSRQGGLNGGAFTATLHRHFLMKCGWQVQV 119

XCJ77335.1 IDRWYTGRLNSWTKAVKTFSFQAVPLPGAFLSRQGGLNGGAFTATLHRHFLMKCGWQVQV 119

XBN89309.1 IDRWYTGRLNSWTKAVKTFSFQAVPLPGAFLSRQGGLNGGAFTATLHRHFLMKCGWQVQV 119

XBN89307.1 IDRWYTGRLNSWTKAVKTFSFQAVPLPGAFLSRQGGLNGGAFTATLHRHFLMKCGWQVQV 119

XBN89295.1 IDRWYTGRLNSWTKAVKTFSFQAVPLPGAFLSRQGGLNGGAFTATLHRHFLMKCGWQVQV 119

XBN89292.1 IDRWYTGRLNSWTKAVKTFSFQAVPLPGAFLSRQGGLNGGAFTATLHRHFLMKCGWQVQV 119

XBN89293.1 IDRWYTGRLNSWTKAVKTFSFQAVPLPGAFLSRQGGLNGGAFTATLHRHFLMKCGWQVQV 119

XBN89294.1 IDRWYTGRLNSWTKAVKTFSFQAVPLPGAFLSRQGGLNGGAFTATLHRHFLMKCGWQVQV 119

XBN89296.1 IDRWYTGRLNSWTKAVKTFSFQAVPLPGAFLSRQGGLNGGAFTATLHRHFLMKCGWQVQV 119

XBN89297.1 IDRWYTGRLNSWTKAVKTFSFQAVPLPGAFLSRQGGLNGGAFTATLHRHFLMKCGWQVQV 119

XBN89301.1 IDRWYTGRLNSWTKAVKTFSFQAVPLPGAFLSRQGGLNGGAFTATLHRHFLMKCGWQVQV 119

XBN89302.1 IDRWYTGRLNSWTKAVKTFSFQAVPLPGAFLSRQGGLNGGAFTATLHRHFLMKCGWQVQV 119

XBN89310.1 IDRWYTGRLNSWTKAVKTFSFQAVPLPGAFLSRQGGLNGGAFTATLHRHFLMKCGWQVQV 119

XBN89313.1 IDRWYTGRLNSWTKAVKTFSFQAVPLPGAFLSRQGGLNGGAFTATLHRHFLMKCGWQVQV 119

XBN89315.1 IDRWYTGRLNSWTKAVKTFSFQAVPLPGAFLSRQGGLNGGAFTATLHRHFLMKCGWQVQV 119

XBN89316.1 IDRWYTGRLNSWTKAVKTFSFQAVPLPGAFLSRQGGLNGGAFTATLHRHFLMKCGWQVQV 119

XBN89317.1 IDRWYTGRLNSWTKAVKTFSFQAVPLPGAFLSRQGGLNGGAFTATLHRHFLMKCGWQVQV 119

XBN89318.1 IDRWYTGRLNSWTKAVKTFSFQAVPLPGAFLSRQGGLNGGAFTATLHRHFLMKCGWQVQV 119

XBN89319.1 IDRWYTGRLNSWTKAVKTFSFQAVPLPGAFLSRQGGLNGGAFTATLHRHFLMKCGWQVQV 119

XCJ77326.1 IDRWYTGRLNSWTKAVKTFSFQAVPLPGAFLSRQGGLNGGAFTATLHRHFLMKCGWQVQV 119

XCJ77327.1 IDRWYTGRLNSWTKAVKTFSFQAVPLPGAFLSRQGGLNGGAFTATLHRHFLMKCGWQVQV 119

XCJ77332.1 IDRWYTGRLNSWTKAVKTFSFQAVPLPGAFLSRQGGLNGGAFTATLHRHFLMKCGWQVQV 119

XCJ77333.1 IDRWYTGRLNSWTKAVKTFSFQAVPLPGAFLSRQGGLNGGAFTATLHRHFLMKCGWQVQV 119

XCJ77334.1 IDRWYTGRLNSWTKAVKTFSFQAVPLPGAFLSRQGGLNGGAFTATLHRHFLMKCGWQVQV 119

QWW20847.1 IDRWYTGRLNSWTKAVKTFSFQAVPLPGAFLSRQGGLNGGAFTATLHRHFLMKCGWQVQV 119

YAJ45130.1 IDRWYTGRLNSWTKAVKTFSFQAVPLPGAFLSRQGGLNGGAFTATLHRHFLMKCGWQVQV 120

YAJ45131.1 IDRWYTGRLNSWTKAVKTFSFQAVPLPGAFLSRQGGLNGGAFTATLHRHFLMKCGWQVQV 119

XBN89298.1 IDRWYTGRLNSWTKAVKTFSFQAVPLPGAFLSRQGGLNGGAFTATLHRHFLMKCGWQVQV 119

XBN89299.1 IDRWYTGRLNSWTKAVKTFSFQAVPLPGAFLSRQGGLNGGAFTATLHRHFLMKCGWQVQV 119

XBN89300.1 IDRWYTGRLNSWTKAVKTFSFQAVPLPGAFLSRQGGLNGGAFTATLHRHFLMKCGWQVQV 119

XBN89303.1 IDRWYTGRLNSWTKAVKTFSFQAVPLPGAFLSRQGGLNGGAFTATLHRHFLMKCGWQVQV 119

XBN89304.1 IDRWYTGRLNSWTKAVKTFSFQAVPLPGAFLSRQGGLNGGAFTATLHRHFLMKCGWQVQV 119

XBN89305.1 IDRWYTGRLNSWTKAVKTFSFQAVPLPGAFLSRQGGLNGGAFTATLHRHFLMKCGWQVQV 119

XBN89306.1 IDRWYTGRLNSWTKAVKTFSFQAVPLPGAFLSRQGGLNGGAFTATLHRHFLMKCGWQVQV 119

XBN89308.1 IDRWYTGRLNSWTKAVKTFSFQAVPLPGAFLSRQGGLNGGAFTATLHRHFLMKCGWQVQV 119

XBN89311.1 IDRWYTGRLNSWTKAVKTFSFQAVPLPGAFLSRQGGLNGGAFTATLHRHFLMKCGWQVQV 119

XBN89314.1 IDRWYTGRLNSWTKAVKTFSFQAVPLPGAFLSRQGGLNGGAFTATLHRHFLMKCGWQVQV 119

XBN89320.1 IDRWYTGRLNSWTKAVKTFSFQAVPLPGAFLSRQGGLNGGAFTATLHRHFLMKCGWQVQV 119

XCJ77328.1 IDRWYTGRLNSWTKAVKTFSFQAVPLPGAFLSRQGGLNGGAFTATLHRHFLMKCGWQVQV 119

XCJ77329.1 IDRWYTGRLNSWTKAVKTFSFQAVPLPGAFLSRQGGLNGGAFTATLHRHFLMKCGWQVQV 119

XCJ77330.1 IDRWYTGRLNSWTKAVKTFSFQAVPLPGAFLSRQGGLNGGAFTATLHRHFLMKCGWQVQV 119

XCJ77336.1 IDRWYTGRLNSWTKAVKTFSFQAVPLPGAFLSRQGGLNGGAFTATLHRHFLMKCGWQVQV 119

XCJ77337.1 IDRWYTGRLNSWTKAVKTFSFQAVPLPGAFLSRQGGLNGGAFTATLHRHFLMKCGWQVQV 119

XCJ77338.1 IDRWYTGRLNSWTKAVKTFSFQAVPLPGAFLSRQGGLNGGAFTATLHRHFLMKCGWQVQV 119

XCJ77339.1 IDRWYTGRLNSWTKAVKTFSFQAVPLPGAFLSRQGGLNGGAFTATLHRHFLMKCGWQVQV 119

XCJ77340.1 IDRWYTGRLNSWTKAVKTFSFQAVPLPGAFLSRQGGLNGGAFTATLHRHFLMKCGWQVQV 119

XCJ77341.1 IDRWYTGRLNSWTKAVKTFSFQAVPLPGAFLSRQGGLNGGAFTATLHRHFLMKCGWQVQV 119

XCJ77343.1 IDRWYTGRLNSWTKAVKTFSFQAVPLPGAFLSRQGGLNGGAFTATLHRHFLMKCGWQVQV 119

UEW62688.1 IDRWYTGRLNSWTKAVKTFSFQAVPLPGAFLSRQGGLNGGAFTATLHRHFLMKCGWQVQV 119

UEW62689.1 IDRWYTGRLNSWTKAVKTFSFQAVPLPGAFLSRQGGLNGGAFTATLHRHFLMKCGWQVQV 119

UEW62690.1 IDRWYTGRLNSWTKAVKTFSFQAVPLPGAFLSRQGGLNGGAFTATLHRHFLMKCGWQVQV 119

UEW62691.1 IDRWYTGRLNSWTKAVKTFSFQAVPLPGAFLSRQGGLNGGAFTATLHRHFLMKCGWQVQV 119

UEW62692.1 IDRWYTGRLNSWTKAVKTFSFQAVPLPGAFLSRQGGLNGGAFTATLHRHFLMKCGWQVQV 119

UEW62693.1 IDRWYTGRLNSWTKAVKTFSFQAVPLPGAFLSRQGGLNGGAFTATLHRHFLMKCGWQVQV 119

QWW20848.1 IDRWYTGRLNSWTKAVKTFSFQAVPLPGAFLSRQGGLNGGAFTATLHRHFLMKCGWQVQV 119

QWW20849.1 IDRWYTGRLNSWTKAVKTFSFQAVPLPGAFLSRQGGLNGGAFTATLHRHFLMKCGWQVQV 119

QWW20850.1 IDRWYTGRLNSWTKAVKTFSFQAVPLPGAFLSRQGGLNGGAFTATLHRHFLMKCGWQVQV 119

************************ ***********************************

QWW20846.1 QCNLTQFHQGALLVAMVPETTLDVKPDGKAKSLQELNEEQWVEMSDDYRTGKNMPFQSLG 179

WBL45177.1 QCNLTQFHQGALLVAMVPETTLDVKPDGKAKSLQELNEEQWVEMSDDYRTGKNMPFQSLG 179

XCJ77342.1 QCNLTQFHQGALLVAMVPETTLDVKPDGKLKSLQELNEEQWVEMSDDYRTGKNMPFQSLG 179

QWW20845.1 QCNLTQFHQGALLVAMVPETTLDVKPDGKAKSLRELNEEQWVEMSDDYRTGKNMPFQSLG 179

XBN89312.1 QCNLTQFHQGALLVAMVPETTLDVKPDGKAKSLQELNEEQWVEMSDDYRTGKNMPFQSLG 179

XCJ77331.1 QCNLTQFHQGALLVAMVPETTLDVKPDGKAKSLQELNEEQWVEMSDDYRTGKNMPFQSLG 179

XCJ77335.1 QCNLTQFHQGALLVAMVPETTLDVKPDGKAKSLQELNEEQWVEMSDDYRTGKNMPFQSLG 179

XBN89309.1 QCNLTQFHQGALLVAMVPETTLDVKPDGKAKSLQELNEEQWVEMSDDYRTGKNMPFQSLG 179

XBN89307.1 QCNLTQFHQGALLVAMVPETTLDVKPDGKAKSXQELNEEQWVEMSDDYRTGKNMPFQSLG 179

XBN89295.1 QCNLTQFHQGALLVAMVPETTLDVKPDGKAKSLQELNEEQWVEMSDDYRTGKNMPYQSLG 179

XBN89292.1 QCNLTQFHQGALLVAMVPETTLDVKPDGKAKSLQELNEEQWVEMSDDYRTGKNMPFQSLG 179

XBN89293.1 QCNLTQFHQGALLVAMVPETTLDVKPDGKAKSLQELNEEQWVEMSDDYRTGKNMPFQSLG 179

XBN89294.1 QCNLTQFHQGALLVAMVPETTLDVKPDGKAKSLQELNEEQWVEMSDDYRTGKNMPFQSLG 179

XBN89296.1 QCNLTQFHQGALLVAMVPETTLDVKPDGKAKSLQELNEEQWVEMSDDYRTGKNMPFQSLG 179

XBN89297.1 QCNLTQFHQGALLVAMVPETTLDVKPDGKAKSLQELNEEQWVEMSDDYRTGKNMPFQSLG 179

XBN89301.1 QCNLTQFHQGALLVAMVPETTLDVKPDGKAKSLQELNEEQWVEMSDDYRTGKNMPFQSLG 179

XBN89302.1 QCNLTQFHQGALLVAMVPETTLDVKPDGKAKSLQELNEEQWVEMSDDYRTGKNMPFQSLG 179

XBN89310.1 QCNLTQFHQGALLVAMVPETTLDVKPDGKAKSLQELNEEQWVEMSDDYRTGKNMPFQSLG 179

XBN89313.1 QCNLTQFHQGALLVAMVPETTLDVKPDGKAKSLQELNEEQWVEMSDDYRTGKNMPFQSLG 179

XBN89315.1 QCNLTQFHQGALLVAMVPETTLDVKPDGKAKSLQELNEEQWVEMSDDYRTGKNMPFQSLG 179

XBN89316.1 QCNLTQFHQGALLVAMVPETTLDVKPDGKAKSLQELNEEQWVEMSDDYRTGKNMPFQSLG 179

XBN89317.1 QCNLTQFHQGALLVAMVPETTLDVKPDGKAKSLQELNEEQWVEMSDDYRTGKNMPFQSLG 179

XBN89318.1 QCNLTQFHQGALLVAMVPETTLDVKPDGKAKSLQELNEEQWVEMSDDYRTGKNMPFQSLG 179

XBN89319.1 QCNLTQFHQGALLVAMVPETTLDVKPDGKAKSLQELNEEQWVEMSDDYRTGKNMPFQSLG 179

XCJ77326.1 QCNLTQFHQGALLVAMVPETTLDVKPDGKAKSLQELNEEQWVEMSDDYRTGKNMPFQSLG 179

XCJ77327.1 QCNLTQFHQGALLVAMVPETTLDVKPDGKAKSLQELNEEQWVEMSDDYRTGKNMPFQSLG 179

XCJ77332.1 QCNLTQFHQGALLVAMVPETTLDVKPDGKAKSLQELNEEQWVEMSDDYRTGKNMPFQSLG 179

XCJ77333.1 QCNLTQFHQGALLVAMVPETTLDVKPDGKAKSLQELNEEQWVEMSDDYRTGKNMPFQSLG 179

XCJ77334.1 QCNLTQFHQGALLVAMVPETTLDVKPDGKAKSLQELNEEQWVEMSDDYRTGKNMPFQSLG 179

QWW20847.1 QCNLTQFHQGALLVAMVPETTLDVKPDGKAKSLQELNEEQWVEMSDDYRTGKNMPFQSLG 179

YAJ45130.1 QCNLTQFHQGALLVAMVPETTLDVKPDGKAKSLQELNEEQWVEMSDDYRTGKNMPFQSLG 180

YAJ45131.1 QCNLTQFHQGALLVAMVPETTLDVKPDGKAKSLQELNEEQWVEMSDDYRTGKNMPFQSLG 179

XBN89298.1 QCNLTQFHQGALLVAMVPETTLDVKPDGKAKSLQELNEEQWVEMSDDYRTGKNMPFQSLG 179

XBN89299.1 QCNLTQFHQGALLVAMVPETTLDVKPDGKAKSLQELNEEQWVEMSDDYRTGKNMPFQSLG 179

XBN89300.1 QCNLTQFHQGALLVAMVPETTLDVKPDGKAKSLQELNEEQWVEMSDDYRTGKNMPFQSLG 179

XBN89303.1 QCNLTQFHQGALLVAMVPETTLDVKPDGKAKSLQELNEEQWVEMSDDYRTGKNMPFQSLG 179

XBN89304.1 QCNLTQFHQGALLVAMVPETTLDVKPDGKAKSLQELNEEQWVEMSDDYRTGKNMPFQSLG 179

XBN89305.1 QCNLTQFHQGALLVAMVPETTLDVKPDGKAKSLQELNEEQWVEMSDDYRTGKNMPFQSLG 179

XBN89306.1 QCNLTQFHQGALLVAMVPETTLDVKPDGKAKSLQELNEEQWVEMSDDYRTGKNMPFQSLG 179

XBN89308.1 QCNLTQFHQGALLVAMVPETTLDVKPDGKAKSLQELNEEQWVEMSDDYRTGKNMPFQSLG 179

XBN89311.1 QCNLTQFHQGALLVAMVPETTLDVKPDGKAKSLQELNEEQWVEMSDDYRTGKNMPFQSLG 179

XBN89314.1 QCNLTQFHQGALLVAMVPETTLDVKPDGKAKSLQELNEEQWVEMSDDYRTGKNMPFQSLG 179

XBN89320.1 QCNLTQFHQGALLVAMVPETTLDVKPDGKAKSLQELNEEQWVEMSDDYRTGKNMPFQSLG 179

XCJ77328.1 QCNLTQFHQGALLVAMVPETTLDVKPDGKAKSLQELNEEQWVEMSDDYRTGKNMPFQSLG 179

XCJ77329.1 QCNLTQFHQGALLVAMVPETTLDVKPDGKAKSLQELNEEQWVEMSDDYRTGKNMPFQSLG 179

XCJ77330.1 QCNLTQFHQGALLVAMVPETTLDVKPDGKAKSLQELNEEQWVEMSDDYRTGKNMPFQSLG 179

XCJ77336.1 QCNLTQFHQGALLVAMVPETTLDVKPDGKAKSLQELNEEQWVEMSDDYRTGKNMPFQSLG 179

XCJ77337.1 QCNLTQFHQGALLVAMVPETTLDVKPDGKAKSLQELNEEQWVEMSDDYRTGKNMPFQSLG 179

XCJ77338.1 QCNLTQFHQGALLVAMVPETTLDVKPDGKAKSLQELNEEQWVEMSDDYRTGKNMPFQSLG 179

XCJ77339.1 QCNLTQFHQGALLVAMVPETTLDVKPDGKAKSLQELNEEQWVEMSDDYRTGKNMPFQSLG 179

XCJ77340.1 QCNLTQFHQGALLVAMVPETTLDVKPDGKAKSLQELNEEQWVEMSDDYRTGKNMPFQSLG 179

XCJ77341.1 QCNLTQFHQGALLVAMVPETTLDVKPDGKAKSLQELNEEQWVEMSDDYRTGKNMPFQSLG 179

XCJ77343.1 QCNLTQFHQGALLVAMVPETTLDVKPDGKAKSLQELNEEQWVEMSDDYRTGKNMPFQSLG 179

UEW62688.1 QCNLTQFHQGALLVAMVPETTLDVKPDGKAKSLQELNEEQWVEMSDDYRTGKNMPFQSLG 179

UEW62689.1 QCNLTQFHQGALLVAMVPETTLDVKPDGKAKSLQELNEEQWVEMSDDYRTGKNMPFQSLG 179

UEW62690.1 QCNLTQFHQGALLVAMVPETTLDVKPDGKAKSLQELNEEQWVEMSDDYRTGKNMPFQSLG 179

UEW62691.1 QCNLTQFHQGALLVAMVPETTLDVKPDGKAKSLQELNEEQWVEMSDDYRTGKNMPFQSLG 179

UEW62692.1 QCNLTQFHQGALLVAMVPETTLDVKPDGKAKSLQELNEEQWVEMSDDYRTGKNMPFQSLG 179

UEW62693.1 QCNLTQFHQGALLVAMVPETTLDVKPDGKAKSLQELNEEQWVEMSDDYRTGKNMPFQSLG 179

QWW20848.1 QCNLTQFHQGALLVAMVPETTLDVKPDGKAKSLQELNEEQWVEMSDDYRTGKNMPFQSLG 179

QWW20849.1 QCNLTQFHQGALLVAMVPETTLDVKPDGKAKSLQELNEEQWVEMSDDYRTGKNMPFQSLG 179

QWW20850.1 QCNLTQFHQGALLVAMVPETTLDVKPDGKAKSLQELNEEQWVEMSDDYRTGKNMPFQSLG 179

***************************** ** :*********************:****

QWW20846.1 TYYRPPNWTWGPNFINPYQVTVFPHQILNARTSTSVDINVPYIGETPTQSSETQNSWTLL 239

WBL45177.1 TYYRPPNWTWGPNYINPYQVTVFPHQILNARTSTSVDISVPYIGETPTQSSETQNSWTLL 239

XCJ77342.1 TYYRPPNWTWGPNFINPYQVTVFPHQILNARTSTSVDISVPYIGETPTQSSETQNSWTLL 239

QWW20845.1 TYYRPPNWTWGPNFINPYQVTVFPHQILNARTSTSVDISVPYIGETPTQSSETQNSWTLL 239

XBN89312.1 TYYRPPNWTWGPNFINPYQVTVFPHQILNARTSTSVDISVPYIGETPTQSSETQNSWTLL 239

XCJ77331.1 TYYRPPNWTWGPNFINPYQVTVFPHQILNARTSTSVDISVPYIGETPTQSSETQNSWTLL 239

XCJ77335.1 TYYRPPNWTWGPNFINPYQVTVFPHQILNARTSTSVDISVPYIGETPTQSSETQNSWTLL 239

XBN89309.1 TYYRPPNWTWGPNFINPYQVTVFPHQILNARTSTSVDISVPYIGETPTQSSETQNSWTLL 239

XBN89307.1 TYYRPPNWTWGPNFINPYQVTVFPHQILNARTSTSVDISVPYIGETPTQSSETQNSWTLL 239

XBN89295.1 TYYRPPNWTWGPNFINPYQVTVFPHQILNARTSTSVDISVPYIGETPTQSSETQNSWTLL 239

XBN89292.1 TYYRPPNWTWGPNFINPYQVTVFPHQILNARTSTSVDISVPYIGETPTQSSETQNSWTLL 239

XBN89293.1 TYYRPPNWTWGPNFINPYQVTVFPHQILNARTSTSVDISVPYIGETPTQSSETQNSWTLL 239

XBN89294.1 TYYRPPNWTWGPNFINPYQVTVFPHQILNARTSTSVDISVPYIGETPTQSSETQNSWTLL 239

XBN89296.1 TYYRPPNWTWGPNFINPYQVTVFPHQILNARTSTSVDISVPYIGETPTQSSETQNSWTLL 239

XBN89297.1 TYYRPPNWTWGPNFINPYQVTVFPHQILNARTSTSVDISVPYIGETPTQSSETQNSWTLL 239

XBN89301.1 TYYRPPNWTWGPNFINPYQVTVFPHQILNARTSTSVDISVPYIGETPTQSSETQNSWTLL 239

XBN89302.1 TYYRPPNWTWGPNFINPYQVTVFPHQILNARTSTSVDISVPYIGETPTQSSETQNSWTLL 239

XBN89310.1 TYYRPPNWTWGPNFINPYQVTVFPHQILNARTSTSVDISVPYIGETPTQSSETQNSWTLL 239

XBN89313.1 TYYRPPNWTWGPNFINPYQVTVFPHQILNARTSTSVDISVPYIGETPTQSSETQNSWTLL 239

XBN89315.1 TYYRPPNWTWGPNFINPYQVTVFPHQILNARTSTSVDISVPYIGETPTQSSETQNSWTLL 239

XBN89316.1 TYYRPPNWTWGPNFINPYQVTVFPHQILNARTSTSVDISVPYIGETPTQSSETQNSWTLL 239

XBN89317.1 TYYRPPNWTWGPNFINPYQVTVFPHQILNARTSTSVDISVPYIGETPTQSSETQNSWTLL 239

XBN89318.1 TYYRPPNWTWGPNFINPYQVTVFPHQILNARTSTSVDISVPYIGETPTQSSETQNSWTLL 239

XBN89319.1 TYYRPPNWTWGPNFINPYQVTVFPHQILNARTSTSVDISVPYIGETPTQSSETQNSWTLL 239

XCJ77326.1 TYYRPPNWTWGPNFINPYQVTVFPHQILNARTSTSVDISVPYIGETPTQSSETQNSWTLL 239

XCJ77327.1 TYYRPPNWTWGPNFINPYQVTVFPHQILNARTSTSVDISVPYIGETPTQSSETQNSWTLL 239

XCJ77332.1 TYYRPPNWTWGPNFINPYQVTVFPHQILNARTSTSVDISVPYIGETPTQSSETQNSWTLL 239

XCJ77333.1 TYYRPPNWTWGPNFINPYQVTVFPHQILNARTSTSVDISVPYIGETPTQSSETQNSWTLL 239

XCJ77334.1 TYYRPPNWTWGPNFINPYQVTVFPHQILNARTSTSVDISVPYIGETPTQSSETQNSWTLL 239

QWW20847.1 TYYRPPNWTWGPNFINPYQVTVFPHQILNARTSTSVDISVPYIGETPTQSSETQNSWTLL 239

YAJ45130.1 TYYRPPNWTWGPNFINPYQVTVFPHQILNARTSTSVDISVPYIGETPTQSSETQNSWTLL 240

YAJ45131.1 TYYRPPNWTWGPNFINPYQVTVFPHQILNARTSTSVDISVPYIGETPTQSSETQNSWTLL 239

XBN89298.1 TYYRPPNWTWGPNFINPYQVTVFPHQILNARTSTSVDISVPYIGETPTQSSETQNSWTLL 239

XBN89299.1 TYYRPPNWTWGPNFINPYQVTVFPHQILNARTSTSVDISVPYIGETPTQSSETQNSWTLL 239

XBN89300.1 TYYRPPNWTWGPNFINPYQVTVFPHQILNARTSTSVDISVPYIGETPTQSSETQNSWTLL 239

XBN89303.1 TYYRPPNWTWGPNFINPYQVTVFPHQILNARTSTSVDISVPYIGETPTQSSETQNSWTLL 239

XBN89304.1 TYYRPPNWTWGPNFINPYQVTVFPHQILNARTSTSVDISVPYIGETPTQSSETQNSWTLL 239

XBN89305.1 TYYRPPNWTWGPNFINPYQVTVFPHQILNARTSTSVDISVPYIGETPTQSSETQNSWTLL 239

XBN89306.1 TYYRPPNWTWGPNFINPYQVTVFPHQILNARTSTSVDISVPYIGETPTQSSETQNSWTLL 239

XBN89308.1 TYYRPPNWTWGPNFINPYQVTVFPHQILNARTSTSVDISVPYIGETPTQSSETQNSWTLL 239

XBN89311.1 TYYRPPNWTWGPNFINPYQVTVFPHQILNARTSTSVDISVPYIGETPTQSSETQNSWTLL 239

XBN89314.1 TYYRPPNWTWGPNFINPYQVTVFPHQILNARTSTSVDISVPYIGETPTQSSETQNSWTLL 239

XBN89320.1 TYYRPPNWTWGPNFINPYQVTVFPHQILNARTSTSVDISVPYIGETPTQSSETQNSWTLL 239

XCJ77328.1 TYYRPPNWTWGPNFINPYQVTVFPHQILNARTSTSVDISVPYIGETPTQSSETQNSWTLL 239

XCJ77329.1 TYYRPPNWTWGPNFINPYQVTVFPHQILNARTSTSVDISVPYIGETPTQSSETQNSWTLL 239

XCJ77330.1 TYYRPPNWTWGPNFINPYQVTVFPHQILNARTSTSVDISVPYIGETPTQSSETQNSWTLL 239

XCJ77336.1 TYYRPPNWTWGPNFINPYQVTVFPHQILNARTSTSVDISVPYIGETPTQSSETQNSWTLL 239

XCJ77337.1 TYYRPPNWTWGPNFINPYQVTVFPHQILNARTSTSVDISVPYIGETPTQSSETQNSWTLL 239

XCJ77338.1 TYYRPPNWTWGPNFINPYQVTVFPHQILNARTSTSVDISVPYIGETPTQSSETQNSWTLL 239

XCJ77339.1 TYYRPPNWTWGPNFINPYQVTVFPHQILNARTSTSVDISVPYIGETPTQSSETQNSWTLL 239

XCJ77340.1 TYYRPPNWTWGPNFINPYQVTVFPHQILNARTSTSVDISVPYIGETPTQSSETQNSWTLL 239

XCJ77341.1 TYYRPPNWTWGPNFINPYQVTVFPHQILNARTSTSVDISVPYIGETPTQSSETQNSWTLL 239

XCJ77343.1 TYYRPPNWTWGPNFINPYQVTVFPHQILNARTSTSVDISVPYIGETPTQSSETQNSWTLL 239

UEW62688.1 TYYRPPNWTWGPNFINPYQVTVFPHQILNARTSTSVDISVPYIGETPTQSSETQNSWTLL 239

UEW62689.1 TYYRPPNWTWGPNFINPYQVTVFPHQILNARTSTSVDISVPYIGETPTQSSETQNSWTLL 239

UEW62690.1 TYYRPPNWTWGPNFINPYQVTVFPHQILNARTSTSVDISVPYIGETPTQSSETQNSWTLL 239

UEW62691.1 TYYRPPNWTWGPNFINPYQVTVFPHQILNARTSTSVDISVPYIGETPTQSSETQNSWTLL 239

UEW62692.1 TYYRPPNWTWGPNFINPYQVTVFPHQILNARTSTSVDISVPYIGETPTQSSETQNSWTLL 239

UEW62693.1 TYYRPPNWTWGPNFINPYQVTVFPHQILNARTSTSVDISVPYIGETPTQSSETQNSWTLL 239

QWW20848.1 TYYRPPNWTWGPNFINPYQVTVFPHQILNARTSTSVDISVPYIGETPTQSSETQNSWTLL 239

QWW20849.1 TYYRPPNWTWGPNFINPYQVTVFPHQILNARTSTSVDISVPYIGETPTQSSETQNSWTLL 239

QWW20850.1 TYYRPPNWTWGPNFINPYQVTVFPHQILNARTSTSVDISVPYIGETPTQSSETQNSWTLL 239

*************:************************.*********************

QWW20846.1 VMVLVPLDYKEGATTDPEITFSVRPTSPYFNGLRNRYTAGTDEEQ 284

WBL45177.1 VMVLVPLDYKEGATTDPEITFSVRPTSPYFNGLRNRFTTGTDEEQ 284

XCJ77342.1 VMVLVPLDYKEGATTDPEITFSVRPTSPYFNGLRNRFTTGTGEEQ 284

QWW20845.1 VMVLVPLDYKEGATTDPEITFSVRPTSPYFNGLRNRYTTGTDEEQ 284

XBN89312.1 VMVLVPLDYKEGATTDPEITFSVRPTSPYFNGLRNRXTTGTDEEQ 284

XCJ77331.1 VMVLVPLDYKEGATTDPEITFSVRPTSPYFNGLRNRXTTGTDEEQ 284

XCJ77335.1 VMVLVPLDYKEGATTDPEITFSVRPTSPYFNGLRNRXTTGTDEEQ 284

XBN89309.1 VMVLVPLDYKEGATTDPEITFSVRPTSPYFNGLRNRFTTGTEEEQ 284

XBN89307.1 VMVLVPLDYKEGATTDPEITFSVRPTSPYFNGLRNRYTTGTDEEQ 284

XBN89295.1 VMVLVPLDYKEGATTDPEITFSVRPTSPYFNGLRNRYTTGTDEEQ 284

XBN89292.1 VMVLVPLDYKEGATTDPEITFSVRPTSPYFNGLRNRYTTGTDEEQ 284

XBN89293.1 VMVLVPLDYKEGATTDPEITFSVRPTSPYFNGLRNRYTTGTDEEQ 284

XBN89294.1 VMVLVPLDYKEGATTDPEITFSVRPTSPYFNGLRNRYTTGTDEEQ 284

XBN89296.1 VMVLVPLDYKEGATTDPEITFSVRPTSPYFNGLRNRYTTGTDEEQ 284

XBN89297.1 VMVLVPLDYKEGATTDPEITFSVRPTSPYFNGLRNRYTTGTDEEQ 284

XBN89301.1 VMVLVPLDYKEGATTDPEITFSVRPTSPYFNGLRNRYTTGTDEEQ 284

XBN89302.1 VMVLVPLDYKEGATTDPEITFSVRPTSPYFNGLRNRYTTGTDEEQ 284

XBN89310.1 VMVLVPLDYKEGATTDPEITFSVRPTSPYFNGLRNRYTTGTDEEQ 284

XBN89313.1 VMVLVPLDYKEGATTDPEITFSVRPTSPYFNGLRNRYTTGTDEEQ 284

XBN89315.1 VMVLVPLDYKEGATTDPEITFSVRPTSPYFNGLRNRYTTGTDEEQ 284

XBN89316.1 VMVLVPLDYKEGATTDPEITFSVRPTSPYFNGLRNRYTTGTDEEQ 284

XBN89317.1 VMVLVPLDYKEGATTDPEITFSVRPTSPYFNGLRNRYTTGTDEEQ 284

XBN89318.1 VMVLVPLDYKEGATTDPEITFSVRPTSPYFNGLRNRYTTGTDEEQ 284

XBN89319.1 VMVLVPLDYKEGATTDPEITFSVRPTSPYFNGLRNRYTTGTDEEQ 284

XCJ77326.1 VMVLVPLDYKEGATTDPEITFSVRPTSPYFNGLRNRYTTGTDEEQ 284

XCJ77327.1 VMVLVPLDYKEGATTDPEITFSVRPTSPYFNGLRNRYTTGTDEEQ 284

XCJ77332.1 VMVLVPLDYKEGATTDPEITFSVRPTSPYFNGLRNRYTTGTDEEQ 284

XCJ77333.1 VMVLVPLDYKEGATTDPEITFSVRPTSPYFNGLRNRYTTGTDEEQ 284

XCJ77334.1 VMVLVPLDYKEGATTDPEITFSVRPTSPYFNGLRNRYTTGTDEEQ 284

QWW20847.1 VMVLVPLDYKEGATTDPEITFSVRPTSPYFNGLRNRYTTGTDEEQ 284

YAJ45130.1 VMVLVPLDYKEGATTDPEITFSVRPTSPYFNGLRNRFTTGTDEE- 284

YAJ45131.1 VMVLVPLDYKEGATTDPEITFSVRPTSPYFNGLRNRFTTGTDEEQ 284

XBN89298.1 VMVLVPLDYKEGATTDPEITFSVRPTSPYFNGLRNRFTTGTDEEQ 284

XBN89299.1 VMVLVPLDYKEGATTDPEITFSVRPTSPYFNGLRNRFTTGTDEEQ 284

XBN89300.1 VMVLVPLDYKEGATTDPEITFSVRPTSPYFNGLRNRFTTGTDEEQ 284

XBN89303.1 VMVLVPLDYKEGATTDPEITFSVRPTSPYFNGLRNRFTTGTDEEQ 284

XBN89304.1 VMVLVPLDYKEGATTDPEITFSVRPTSPYFNGLRNRFTTGTDEEQ 284

XBN89305.1 VMVLVPLDYKEGATTDPEITFSVRPTSPYFNGLRNRFTTGTDEEQ 284

XBN89306.1 VMVLVPLDYKEGATTDPEITFSVRPTSPYFNGLRNRFTTGTDEEQ 284

XBN89308.1 VMVLVPLDYKEGATTDPEITFSVRPTSPYFNGLRNRFTTGTDEEQ 284

XBN89311.1 VMVLVPLDYKEGATTDPEITFSVRPTSPYFNGLRNRFTTGTDEEQ 284

XBN89314.1 VMVLVPLDYKEGATTDPEITFSVRPTSPYFNGLRNRFTTGTDEEQ 284

XBN89320.1 VMVLVPLDYKEGATTDPEITFSVRPTSPYFNGLRNRFTTGTDEEQ 284

XCJ77328.1 VMVLVPLDYKEGATTDPEITFSVRPTSPYFNGLRNRFTTGTDEEQ 284

XCJ77329.1 VMVLVPLDYKEGATTDPEITFSVRPTSPYFNGLRNRFTTGTDEEQ 284

XCJ77330.1 VMVLVPLDYKEGATTDPEITFSVRPTSPYFNGLRNRFTTGTDEEQ 284

XCJ77336.1 VMVLVPLDYKEGATTDPEITFSVRPTSPYFNGLRNRFTTGTDEEQ 284

XCJ77337.1 VMVLVPLDYKEGATTDPEITFSVRPTSPYFNGLRNRFTTGTDEEQ 284

XCJ77338.1 VMVLVPLDYKEGATTDPEITFSVRPTSPYFNGLRNRFTTGTDEEQ 284

XCJ77339.1 VMVLVPLDYKEGATTDPEITFSVRPTSPYFNGLRNRFTTGTDEEQ 284

XCJ77340.1 VMVLVPLDYKEGATTDPEITFSVRPTSPYFNGLRNRFTTGTDEEQ 284

XCJ77341.1 VMVLVPLDYKEGATTDPEITFSVRPTSPYFNGLRNRFTTGTDEEQ 284

XCJ77343.1 VMVLVPLDYKEGATTDPEITFSVRPTSPYFNGLRNRFTTGTDEEQ 284

UEW62688.1 VMVLVPLDYKEGATTDPEITFSVRPTSPYFNGLRNRFTTGTDEEQ 284

UEW62689.1 VMVLVPLDYKEGATTDPEITFSVRPTSPYFNGLRNRFTTGTDEEQ 284

UEW62690.1 VMVLVPLDYKEGATTDPEITFSVRPTSPYFNGLRNRFTTGTDEEQ 284

UEW62691.1 VMVLVPLDYKEGATTDPEITFSVRPTSPYFNGLRNRFTTGTDEEQ 284

UEW62692.1 VMVLVPLDYKEGATTDPEITFSVRPTSPYFNGLRNRFTTGTDEEQ 284

UEW62693.1 VMVLVPLDYKEGATTDPEITFSVRPTSPYFNGLRNRFTTGTDEEQ 284

QWW20848.1 VMVLVPLDYKEGATTDPEITFSVRPTSPYFNGLRNRFTTGTDEEQ 284

QWW20849.1 VMVLVPLDYKEGATTDPEITFSVRPTSPYFNGLRNRFTTGTDEEQ 284

QWW20850.1 VMVLVPLDYKEGATTDPEITFSVRPTSPYFNGLRNRFTTGTDEEQ 284

************************************ *:** **

**Supplementary Table 1.** Multiple sequence alignment of the SVA VP2 protein. The NCBI virus database was used to collect 485 accession numbers corresponding to deposited protein sequences of the SVA virus open reading frame polypeptide. Python code was written to extract the VP2 protein sequences from the SVA polypeptides only if the GenBank record explicitly annotates the CDS or mature peptide feature within its qualifiers. No inference of the VP2 protein was used based on sequence motifs or length. Total VP2 protein sequences identified = 62. FASTA formatted SVA VP2 sequences were subject to multiple sequence alignment using Clustal Omega (1.2.4) (<https://doi.org/10.1093/nar/gkae241>). The 62 VP2 sequences share 96.7% amino acid identities with a similarity of 97.1%. The SVA VP2 proteins represented in this expanded alignment cohort further support the highly similar VP2 protein within the SVA genus and support broad antibody cross reactivity. Identical amino acids in the cohort indicated by asterisk (*). All VP2 protein sequences evaluated have identical amino acids at the core binding residues corresponding to the 7B3 MAb epitope (rectangles).
