# Supplementary material for: VP2-targeted sandwich ELISA (sELISA) enables direct detection of Senecavirus A (SVA)
Source: J Virol. 2026 May 12;100(6):e00571-26. doi: 10.1128/jvi.00571-26 (PMC13289164; doi:10.1128/jvi.00571-26)
Supplement: Table S2 — Immunoassay binding properties of VP2 antibodies. [file jvi.00571-26-s0006.docx]

| **MAbs** | **iELISA**  **VP2** | **cELISA**  **Btn-VP2** | **iELISA**  **SVA-infected**  **CM** |
| --- | --- | --- | --- |
| 1E2 | + | - | - |
| **2D1** | **+++** | **+++** | **+++** |
| 2G3 | + | +++ | - |
| 4B7 | ++ | +++ | - |
| 5A7 | ++ | - | - |
| 5A8 | +++ | + | - |
| 5G3 | + | +++ | - |
| **7B3** | **+++** | **+++** | **+++** |
| 7C4 | ++ | ++ | - |
| 9A3 | ++ | + | - |
| 9E4 | + | - | - |
| 9H2 | + | - | - |
| 10F3 | + | +++ | - |
| 10G4 | + | - | - |

**Supplementary Table 2.** Immunoassay binding properties of antibodies generated against the SVA VP2 protein. A cohort of 14 hybridoma cell lines producing anti-VP2 MAb were evaluated using a differential immunoassay screening strategy to identify those with optimal performance characteristics. Initial screening and selection were performed by indirect ELISA (iELISA) using immobilized recombinant VP2 and hybridoma cell conditioned media (CM) with an anti-mouse gamma chain-HRP used in detection. Secondary screening and selection were performed using a capture ELISA (cELISA) where immobilized anti-mouse Fc was used to capture MAb from CM, following by solution capture of a biotinylated recombinant VP2 with detection using an avidin-HRP conjugate. NCI-H1299 cell monolayers were subject to infection with SVA for 2d and cells harvested, homogenized and clarified with protein extract immobilized and incubated with MAbs, followed by detection with an anti-mouse gamma chain-HRP conjugate (iELISA). Only MAbs capable of detection in all three immunoassays were subject to pairwise sandwich ELISA (sELISA) evaluation for the detection of VP2 and virus.
