# Supplementary material for: VP2-targeted sandwich ELISA (sELISA) enables direct detection of Senecavirus A (SVA)
Source: J Virol. 2026 May 12;100(6):e00571-26. doi: 10.1128/jvi.00571-26 (PMC13289164; doi:10.1128/jvi.00571-26)
Supplement: Table S3A — Multiple sequence alignment of picornavirus VP2 proteins. [file jvi.00571-26-s0007.docx]

Hepatovirus_HAV_VP2|AKI05745.1|positions_24-245 DIEEEQMIQSVDRTAVTGASYFTSVDQSSVHT----AEVGSHQVEPLRTSVDKPGSKRTQ 56

Aphthovirus_FMDV_O1_VP2|AAF09193.1|positions_287-503 DKKTEETTLLEDRILTTRNGHTTSTTHSSVGVTYGYATAEDFVSGPNTSGLE---TRVAQ 57

Senecavirus_SVA_SD15_26_VP2|APY18927.1|positions_151-434 DHNTEEMENSADRVITQTAGNTAINTQSSLGVLCAYVEDPTK-SDPPSSSTDQPTTTFTA 59

Cardiovirus_EMCV_VP2|CAA60776.1|positions_138-393 DQNTEEMENLSDRVLQDTAGNTVTNTQSTVGRLVGYGAVHDG--EHPASCADTASEKILA 58

Enterovirus_SVDV_D68_VP2|ABL61317.1|positions_70-317 -SPSAEACGYSDRVLQLKLGNSAIVTQEAANYCCAYGEWPNYLPDHEAVAIDKPTQPETA 59

: ** . . :.: :

Hepatovirus_HAV_VP2|AKI05745.1|positions_24-245 GEKFFLIHSADWLTTHALFHEVAKLDVVKLL-----YNEQFAVQGLLRYHTYARFGIEIQ 111

Aphthovirus_FMDV_O1_VP2|AAF09193.1|positions_287-503 AERFFKTHLFDWVPSDPFGRCHLL-ELP--------TEHKGVYGSLTDSYAYMRNGWDVE 108

Senecavirus_SVA_SD15_26_VP2|APY18927.1|positions_151-434 IDRWYTGRLNSWTKAVKTFSFQAV-PLPGAFLSRQGGLNGGAFTATLHRHFLMKCGWQVQ 118

Cardiovirus_EMCV_VP2|CAA60776.1|positions_138-393 VERYYTFKVNDWTSTQKPFEYIRI-PLPHVLS----GEDGGVFGAALRRHYLVKTGWRVQ 113

Enterovirus_SVDV_D68_VP2|ABL61317.1|positions_70-317 TDRFYTLKSVKWEAGSTGWWWK----LPDA------LNNIGMFGQNVQHHYLYRSGFLIH 109

:::: : .* : . : : * :.

Hepatovirus_HAV_VP2|AKI05745.1|positions_24-245 VQINPTPFQQGGLICAMVPGDQS------------------------------------- 134

Aphthovirus_FMDV_O1_VP2|AAF09193.1|positions_287-503 VTAVGNQFNGGCLLVAMVPELSFS------------------------------------ 132

Senecavirus_SVA_SD15_26_VP2|APY18927.1|positions_151-434 VQCNLTQFHQGALLVAMVPETTLDVKPDGKAKSLQELNEEQWVEMSDDYRTGKNMPFQSL 178

Cardiovirus_EMCV_VP2|CAA60776.1|positions_138-393 VQCNASQFHAGSLLVFMAPEYPTLDAF---------AMDNRWSKD--NLPNGTKTQTNRK 162

Enterovirus_SVDV_D68_VP2|ABL61317.1|positions_70-317 VQCNATKFHQGALLVVAIPEHQRGAYNTNTSPEFNDIM------------K-----GEEG 152

* . *: * *: *

Hepatovirus_HAV_VP2|AKI05745.1|positions_24-245 --------------YGSIASLTVYPHGLLNCNINNVVRIKVPFIYTRGAYHFKDPQYPVW 180

Aphthovirus_FMDV_O1_VP2|AAF09193.1|positions_287-503 --------------KRELYQFTFFPHQFIKPSNEMTAHITVPFVGVNRYDQY--KTHKPW 176

Senecavirus_SVA_SD15_26_VP2|APY18927.1|positions_151-434 GTYYRPPNWTWGPNFINPYQVTVFPHQILNARTSTSVDISVPYIGETPTQSS--ETQNSW 236

Cardiovirus_EMCV_VP2|CAA60776.1|positions_138-393 GP--------FAMDHQNFWQWTLYPHQFLNLRTNTTVDLEVPYVNIAPTSSW--TQHASW 212

Enterovirus_SVDV_D68_VP2|ABL61317.1|positions_70-317 GTFNHPYVL---DDGTSLACATIFPHQWINLRTNNSATIVLPWMNAAPMDFP--LRHNQW 207

. *.:** :: . . : :*:: *

Hepatovirus_HAV_VP2|AKI05745.1|positions_24-245 ELTIRVWSELNIGTGTSAYTSLNVLARFTDLELHGLTPLSTQ------ 222

Aphthovirus_FMDV_O1_VP2|AAF09193.1|positions_287-503 TLVVMVVAPLTVNNEGAPQIKVYANIAPTSVHVAGEFPSKE------- 217

Senecavirus_SVA_SD15_26_VP2|APY18927.1|positions_151-434 TLLVMVLVPLDYKEGATTDPEITFSVRPTSPYFNGLRNRFTTGTDEEQ 284

Cardiovirus_EMCV_VP2|CAA60776.1|positions_138-393 TLVIAVVAPLTYSTGASTSLDITASIQPVRPVFNGLRHETLSRQ---- 256

Enterovirus_SVDV_D68_VP2|ABL61317.1|positions_70-317 TLAIIPVVPLGTRTMS-SMVPITVSIAPMCCEFNGLRHAITQ------ 248

* : * : . *

**Supplementary Table 3A.** Multiple sequence alignment of picornavirus VP2 proteins using Clustal O (1.2.4). Identical amino acid resides indicated by asterik (*).
