# Supplementary material for: VP2-targeted sandwich ELISA (sELISA) enables direct detection of Senecavirus A (SVA)
Source: J Virol. 2026 May 12;100(6):e00571-26. doi: 10.1128/jvi.00571-26 (PMC13289164; doi:10.1128/jvi.00571-26)
Supplement: Table S3B — Pairwise alignment of VP2 protein from SVA and FMDV. [file jvi.00571-26-s0008.docx]

APY18927 1 DHNTEEMENSADRVITQTAGNTAINTQSSLGVLCAY--VEDPTKSDPPSS 48

|..|||.....||::|...|:|...|.||:||...| .|| ..|.|.:|

AAF09193 1 DKKTEETTLLEDRILTTRNGHTTSTTHSSVGVTYGYATAED-FVSGPNTS 49

APY18927 49 STDQPTTTFTAIDRWYTGRLNSWTKAVKTFSFQAVPLPGAFLSRQGGLNG 98

..: |.....:|::...|..|..:........:.||.. :.

AAF09193 50 GLE---TRVAQAERFFKTHLFDWVPSDPFGRCHLLELPTE--------HK 88

APY18927 99 GAFTATLHRHFLMKCGWQVQVQCNLTQFHQGALLVAMVPETTLDVKPDGK 148

|.:.:....:..|:.||.|:|.....||:.|.||||||||.:.

AAF09193 89 GVYGSLTDSYAYMRNGWDVEVTAVGNQFNGGCLLVAMVPELSF------- 131

APY18927 149 AKSLQELNEEQWVEMSDDYRTGKNMPFQSLGTYYRPPNWTWGPNFINPYQ 198

|.:|| ||

AAF09193 132 --SKREL-----------------------------------------YQ 138

APY18927 199 VTVFPHQILNARTSTSVDISVPYIGETPTQSSETQNSWTLLVMVLVPLDY 248

.|.||||.:......:..|:||::|.......:|...|||:|||:.||..

AAF09193 139 FTFFPHQFIKPSNEMTAHITVPFVGVNRYDQYKTHKPWTLVVMVVAPLTV 188

APY18927 249 -KEGATTDPEITF--SVRPTSPYFNGLRNRFTTGTDEEQ 284

.||| |:|.. ::.|||.:..| .|.:..

AAF09193 189 NNEGA---PQIKVYANIAPTSVHVAG---EFPSKE---- 217

**Supplementary Table 3B**. Pairwise alignment of VP2 protein from SVA and FMDV (EMBOSS Needle DOI: 10.1093/nar/gkae241). The VP2 protein from Senecavirus SVA/SD15-26 (Accession No. APY18927) (residues 151-434) was aligned with the VP2 protein from Aphthovirus FMDV/01 (Accession No. AAF09193) (residues 287-503). Matrix: EBLOSUM62, Identity: 76/289 (26.3%), Similarity: 109/289 (37.7%), Gaps: 77/289 (26.6%), Score: 222.5. Vertical lines indicate identical residues.
