# Supplementary material for: VP2-targeted sandwich ELISA (sELISA) enables direct detection of Senecavirus A (SVA)
Source: J Virol. 2026 May 12;100(6):e00571-26. doi: 10.1128/jvi.00571-26 (PMC13289164; doi:10.1128/jvi.00571-26)
Supplement: Table S4 — SVA VP2 peptides used for epitope mapping. [file jvi.00571-26-s0009.docx]

| No. | SVA VP2 Peptides |
| --- | --- |
| 1 | MDHNTEEMENSADRV |
| 2 | SADRVITQTAGNTAI |
| 3 | GNTAINTQSSLGVLC |
| 4 | LGVLCAYVEDPTKSD |
| 5 | PTKSDPPSSSTDQPT |
| 6 | TDQPTTTFTAIDRWY |
| 7 | IDRWYTGRLNSWTKA |
| 8 | SWTKAVKTFSFQAVP |
| 9 | FQAVPLPGAFLSRQG |
| 10 | LSRQGGLNGGAFTAT |
| 11 | AFTATLHRHFLMKCG |
| 12 | LMKCGWQVQVQCNLT |
| 13 | QCNLTQFHQGALLVA |
| 14 | ALLVAMVPETTLDVK |
| 15 | TLDVKPDGKAKSLQE |
| 16 | KSLQELNEEQWVEMS |
| 17 | WVEMSDDYRTGKNMP |
| 18 | GKNMPFQSLGTYYRP |
| 19 | TYYRPPNWTWGPNFI |
| 20 | GPNFINPYQVTVFPH |
| 21 | TVFPHQILNARTSTS |
| 22 | RTSTSVDISVPYIGE |
| 23 | PYIGETPTQSSETQN |
| 24 | SETQNSWTLLVMVLV |
| 25 | VMVLVPLDYKEGATT |
| 26 | EGATTDPEITFSVRP |
| 27 | FSVRPTSPYFNGLRN |
| 28 | NGLRNRFTTGTDEEQ |
| 15 | TLDVKPDGKAKSLQE |
| 16 | KSLQELNEEQWVEMS |
| 17 | WVEMSDDYRTGKNMP |
| 18 | GKNMPFQSLGTYYRP |
| 19 | TYYRPPNWTWGPNFI |
| 20 | GPNFINPYQVTVFPH |
| 21 | TVFPHQILNARTSTS |
| 22 | RTSTSVDISVPYIGE |
| 23 | PYIGETPTQSSETQN |
| 24 | SETQNSWTLLVMVLV |
| 25 | VMVLVPLDYKEGATT |
| 26 | EGATTDPEITFSVRP |
| 27 | FSVRPTSPYFNGLRN |
| 28 | NGLRNRFTTGTDEEQ |

**Supplemental Table 4.** Synthetic peptides of the SVA VP2 protein. A series of 28 sequential peptides, each 15 amino acids in length with a 5 amino acid sequence overlap of the previous peptide were synthesized with an N-terminal biotin-Ahx (Genscript, NJ) and confirmed by mass spectrometry. Peptides were dissolved in water, NMP, 3% NH4 or DMSO to 10mM then sonicated briefly at 37ºC. High-binding 96-well microplates containing immobilized streptavidin (Millipore; 2µg/mL) were blocking in 10% NFDM in TBST then incubated with 100µL of each peptide diluted to 25µM in TBST overnight at room temperature. Direct ELISA were performed for each peptide in triplicate with either the 7B3-HRP or 2D1-HRP (1µg/mL) SVA VP MAb. MAb binding to each peptide was determined by chemiluminescent detection (Ultra ECL; Neogen) using a Victor X3 luminometer (Perkin-Elmer) and reported in counts per second (CPS). The 7B3 MAb bound peptides 16 and 23 (P=<0.001). No significant binding was observed with the 2D1 MAb to any of the peptides.
